# Supplementary material for: Breaking the cold chain: solutions for room temperature preservation of mosquitoes leading to high quality reference genomes
Source: Gigascience. 2026 May 22;15:giag061. doi: 10.1093/gigascience/giag061 (PMC13221836; doi:10.1093/gigascience/giag061)
Supplement: giag061_GIGA-D-25-00389_revision_1 [file giag061_giga-d-25-00389_revision_1.pdf]

GigaScience

Breaking the cold chain: solutions for room temperature preservation of mosquitoes leading to high quality reference genomes

--Manuscript Draft--

**Manuscript Number:**	GIGA-D-25-00389R1
**Full Title:**	Breaking the cold chain: solutions for room temperature preservation of mosquitoes leading to high quality reference genomes
**Article Type:**	Technical Note
**Funding Information:**	Wellcome Trust (220540/Z/20/A) — Dr Mara K. N. Lawniczak<br>Bill and Melinda Gates Foundation (INV-009760) — Dr Mara K. N. Lawniczak
**Abstract:**	Background<br>The Earth BioGenome Project (EBP) is a global endeavour to produce reference genomes for all described eukaryotic species. The majority of described species are arthropods, which tend to be small and require taxonomic expertise to identify to species level. Therefore, the ability to collect and preserve specimens in a suitable way for long read and Hi-C data generation using very simple approaches with minimal infrastructure is certain to be important in scaling up reference genome generation. Field collections requiring dry ice or dry shippers can be logistically challenging to arrange, are notoriously expensive, and DNA degrades rapidly if ultra-cold temperature is not maintained, which is devastating given how expensive and time consuming field work can be.<br>Findings<br>Using Anopheles mosquitoes as an insect representative we evaluate how well different preservation liquids protect high molecular weight DNA, RNA, and nuclei for Hi-C at room temperature when mosquitoes are held intact versus slightly squished. We find that squished samples stored in 100% ethanol and Allprotect held at room temperature for one week result in excellent preservation of both high molecular weight DNA and nuclei for Hi-C. Other tested buffers, including RNAlater, EDTA at several pHs, and DMSO Salt Solution (DESS) performed satisfactorily for long read data generation and RNA retrieval, but less ideally for Hi-C, which may have bigger negative impacts when aiming to generate data for organisms with larger genomes.<br>Conclusions<br>We present multiple viable options for room temperature collection and/or shipment for arthropod samples, with direct suggestions for shipment and storage of mosquitoes and similarly sized specimens. Further exploration across a broader range of species will hopefully enable cheaper and more widely available reference genome generation globally.
**Corresponding Author:**	Petra Korlević, PhD<br>Wellcome Sanger Institute<br>Hinxton, Cambridgeshire UNITED KINGDOM
**Corresponding Author Secondary Information:**	
**Corresponding Author's Institution:**	Wellcome Sanger Institute
**Corresponding Author's Secondary Institution:**	
**First Author:**	Petra Korlević
**First Author Secondary Information:**	
**Order of Authors:**	Petra Korlević
	Fiona Teltscher
	Edel Sheerin
	Alex Makunin

Powered by Editorial Manager® and ProduXion Manager® from Aries Systems Corporation

	Mara K. N. Lawniczak
**Order of Authors Secondary Information:**	
**Response to Reviewers:**	- We thank the reviewers and editor for their insightful comments, and have included most of their suggestions into the manuscript. For the suggestions that require additional labwork we have hopefully made it clear why that is out of the scope of this paper. All new sentences and paragraphs are highlighted with red text in the resubmission. The formatting has also been updated to include several paragraphs that are mandatory in a GigaScience Technical Note publication. Below we go through each comment separately.<br><br>Reviewer 1:<br>This study uses Anopheles coluzzii as a model to systematically evaluate preservation methods for generating high-quality genomic and Hi-C data, addressing a question of clear practical relevance. The development of approaches that eliminate the need for cold-chain transport is a particularly important step forward for field-based genomics, where maintaining low temperatures is often logistically difficult. In this context, the work provides useful experimental evidence and practical guidance with broad potential value. That said, several aspects of the manuscript would benefit from further clarification and refinement, as outlined below :<br>1. The study is conducted exclusively on Anopheles coluzzii under controlled laboratory conditions. While the results are promising, the extent to which these preservation strategies can be applied to other arthropods remains unclear. Given the substantial diversity in cuticle structure, body size, and physiological properties across taxa, the authors should more explicitly discuss the limitations of extrapolating their findings. Inclusion of additional taxa would be ideal; alternatively, a more thorough discussion of potential constraints would improve the manuscript.<br>- We have now added a small paragraph explaining the limitations of the result interpretation when applying these methods to any other arthropod species. As a lab focused on mosquitoes with an in house An. coluzzii colony, it was the easiest and most minimally variable organism we could use across all tests to minimize internal sample bias. We have since sequenced several reference genomes using the above approach for Anopheles samples that were sent squished at room temperature, they are still going through curation but will be published soon as genome notes.<br><br>2. The evaluation of Hi-C data quality is primarily based on scaffolding performance, including analyses using both high-quality and fragmented assemblies. However, the Hi-C datasets were generated at very high sequencing depth (>100×), which may obscure differences among preservation treatments by compensating for variations in data quality. The authors should clarify how they distinguish genuine preservation effects from depth-related compensation. Additional analyses based on downsampled Hi-C datasets would provide a more realistic assessment of performance under typical sequencing conditions.<br>- Hi-C sequencing was indeed done at very high coverage to ensure saturation with long-range interactions. In order to assess Hi-C quality, we have added plots of fragment separation frequencies in log-scale (new figure, now named Fig. 5). These plots highlight better performance for ethanol and AllProtect treated samples, reduction of long-range interaction capture in RNAlater, and dramatic underperforming DESS and EDTA. Note that these patterns are also reflected in scaffolding performance in most complex 10-20% of mosquito genomes representing repeat rich heterochromatin (black bars in Fig. 6).<br><br>3. I am particularly interested in the chromosome anchoring rates observed in the Hi-C scaffolding analyses. It would be valuable for the authors to clarify whether Hi-C data generated from different preservation methods lead to differences in chromosome anchoring efficiency, as this is a key indicator of scaffolding quality.<br>- We left the Hi-C scaffolded plots (now Fig. 6) exactly as they came out of a standard routine pipeline on purpose to highlight which buffers made it possible to get nicely scaffolded expected chromosomal structures in An. coluzzii. At this stage samples would have gone for curation, but we wanted to show which one performed well and would require minimal curation from the get-go. To make the interpretations a bit easier, we now added the % of reads that fall into a chromosomal scaffold for each PacBio and Hi-C combination in the upper right corner of the scaffold plots. This percentage is roughly estimated from the sizes of scaffolds that span at least one

chromosome arm, and showcase how much unscaffolded “shrapnel” is left.

4. The manuscript indicates that sequencing data are not yet publicly available due to their inclusion in a larger ENA project. In line with journal policies, the authors should provide a clear plan for data release, including expected timelines and accession numbers where possible. Furthermore, additional methodological details, particularly regarding genome assembly parameters and Hi-C data processing, would improve reproducibility and transparency.
- Since the manuscript’s submission all raw data has been made available in their specific ENA study (PRJEB98990), and all processed data have been uploaded as part of the review process into a GigaDB study.

5. In Table 1, the inclusion of ULI scaffolding appears somewhat abrupt, as ULI sequencing is not sufficiently introduced or contextualized prior to its presentation in the table. Although its role becomes clearer in the Results section, readers may find it difficult to fully understand its relevance at this stage. The authors may consider introducing ULI sequencing earlier in the Methods or Results, or providing a brief explanation in the table caption, to improve clarity and ensure a more coherent presentation.
- We have now added a paragraph in the Methods section, DNA extraction and long read PacBio sequencing subsection, explaining in more detail on how the ULI sample was prepared and sequenced, as well as the reasoning for its inclusion.

Reviewer 2:
This manuscript presents a systematic evaluation of preservation methods for generating high-quality genomic and Hi-C data using Anopheles coluzzii as a model organism. The study addresses an important practical challenge in genomics, particularly for field-based sample collection where optimal preservation conditions are often difficult to achieve. The experimental design is generally well-structured, and the comparison across multiple preservation treatments provides useful insights for the community.
However, several aspects of the study require further clarification and improvement. In particular, concerns remain regarding the generalizability of the findings beyond the focal species, the robustness of the statistical analyses, and the interpretation of Hi-C results under very high sequencing coverage. Additionally, issues related to data availability and methodological transparency should be addressed to ensure reproducibility. Addressing these points would substantially strengthen the manuscript.
1. The study is conducted exclusively on Anopheles coluzzii under controlled laboratory conditions. While the results are promising, the applicability of these preservation strategies to other arthropods remains unclear. Given the diversity in cuticle structure, body size, and physiology across taxa, the authors should clarify the extent to which their findings can be generalized. Inclusion of additional taxa or a more explicit discussion of limitations would strengthen the manuscript.
- Answered in Reviewer 1. question 1.

2. The evaluation of Hi-C data quality is based on scaffolding performance, including analyses using both high-quality and fragmented assemblies. However, the Hi-C datasets were generated at very high coverage (>100×), which may mask differences in preservation efficiency. The authors should clarify how they distinguish true preservation effects from sequencing depth-related compensation. Additional analyses using downsampled Hi-C data would provide a more realistic assessment of performance under typical conditions.
- Answered in Reviewer 1. question 2.

3. I am particularly interested in the chromosome anchoring rate of the genome assemblies in the Hi-C scaffolding analysis. It would be valuable for the authors to clarify whether Hi-C data generated using different preservation methods result in differences in chromosome anchoring efficiency.
- Answered in Reviewer 1. question 3.

4. The manuscript indicates that sequencing data are not yet publicly available due to their inclusion in a larger ENA project. In line with journal policies, the authors should provide a clear plan for data release, including expected timelines and accession numbers if available. Furthermore, greater detail in the Methods section—particularly

Powered by Editorial Manager® and ProduXion Manager® from Aries Systems Corporation

regarding assembly parameters and Hi-C processing—would improve reproducibility.
- Answered in Reviewer 1. question 4.

5. The column headers “Self scaffolding” and “ULI scaffolding” in Table 1 are not sufficiently clear, making it difficult for readers to fully understand the intended meaning.
- We have now modified the table legend to include a more detailed description of the two column heathers, as well as a paragraph in the Methods section describing the ULI sample in better detail.

Additional reviewer reports:
Reviewer #1: This is a straightforward and valuable study. Demonstrating that breaking the cold chain is feasible has strong potential to transform how genomes can be obtained across much of biodiversity. In my view, the broader significance of this advance is somewhat underemphasized in the Introduction (Really just a short mention around lines 72-75). Based on firsthand experience in large field campaigns designed specifically to generate genomes from very small insects, I can attest that much of arthropod biodiversity (especially within small-bodied, hyperdiverse "dark taxa") stands to benefit substantially from approaches like this. The practical importance of scalable, field-friendly preservation solutions cannot be overstated.
- Overall, the study is well designed, clearly presented, and addresses a real logistical bottleneck in biodiversity genomics. The conclusions are generally well supported by the data presented. I have a few suggestions that I believe would further strengthen the manuscript and increase its practical value and reproducibility.

Major / Substantive Comments
1. Framing and impact
As above, I recommend strengthening the Introduction's framing of downstream biodiversity applications, especially for small-bodied and taxonomically challenging groups where cold-chain logistics are often the primary limiting factor. The method has particularly high relevance for large-scale efforts targeting hyperdiverse insect groups ("dark taxa"), and this applied significance could be more explicitly highlighted to broaden the paper's audience and appeal.
- We thank the reviewer for their enthusiastic comments to our manuscript. We have now added a few more sentences in the Introduction and Discussion sections highlighting the importance of room temperature storage and shipment methods for arthropod and dark taxa studies.

2. Practical protocol clarity and reproducibility
Because this method is likely to be adopted by field teams, including non-specialists, it would be very helpful to include a concise protocol-style summary or workflow outlining the recommended handling steps, specimen size considerations, and timing constraints. A short practical guide or decision framework would improve reproducibility and uptake.
- The detailed description (with images) on how to collect and squish specimens was published separately in a protocols.io SOP, and we have now made that more explicitly clear in the Methods section.

Relatedly, laying out any known tolerance ranges in this fashion would strengthen the paper, for example, how sensitive outcomes are to delays in processing, temperature variation, or specimen size differences under field conditions.
- While we have not tested this on non-Anophelines, we have since received and sequenced several Anopheles species collected and sent from wild caught individuals, which are currently in genome curation and will be published as genome notes in the near future. We unfortunately have not done more extensive testing on storage times and sizes, but since we have adopted this storage and shipment approach on real samples already we are passively able to track this through possible sample failure or underperformance in the future.

3. Preservation benchmarking
I suggest including a summary comparison (table or figure) of yield/quality metrics across preservation treatments relative to standard cold-chain approaches. This would make performance differences easier for readers to interpret and apply.
- We have now added Table 3 in the Discussion section which briefly summarizes

Powered by Editorial Manager® and ProduXion Manager® from Aries Systems Corporation

success across our storage solutions and extraction/sequencing approach (PacBio sequencing, Hi-C sequencing, RNA extraction) and the final rating. This should make the discussion paragraph much easier to follow and for other researchers to decide on what solution to try themselves.

4. Voucher integrity and downstream taxonomic usability
Given that many target organisms will come from taxonomically difficult groups, a short discussion of voucher integrity after treatment would be valuable. Guidance on expected morphological preservation and suitability for downstream taxonomic work would significantly increase the method's usefulness for specimen-based biodiversity genomics workflows.
- As this method is extremely destructive, taxonomy should be performed initially and any valuable body part (such as mouth parts or genitalia) removed prior to tissue grinding. Because of that we were not focused on assessing morphological preservation in the specified storage solutions and subsequent freezer storage. We do highlight in the last sentence of the Discussion section that further work should be done on long term and better storage of samples for morphology, proteins and metabolites, even if they were out of scope for our manuscript.

5. Methodological Clarification
The instruction to "lightly squish" specimens to compromise the cuticle is practical, but could benefit from additional detail. Does the location of compression affect outcomes? For example, is thoracic compression preferable (to access muscle tissue), or is abdominal disruption sufficient? This may seem like a fine detail, but it matters in practice, particularly because damage to thoracic characters or terminalia can reduce taxonomic value. If there is an optimal or recommended compression location, it would be useful to specify it.
- As mentioned in answer 2, we have a fully detailed protocols.io SOP on how to perform the squish approach with images, we now have a sentence highlighting this in the Methods section.

Editor Comments:
To meet the journal’s data availability requirements and ensure the reproducibility of your research findings, we kindly request that you add a Data Availability section in the revised version of your manuscript.
- We have now added a Data Availability paragraph and information to each ENA raw datafile in the supplementary table. While we do not have access to the GigaDB repository to add a reference to it, we suspect these will become available once the paper is out? We have also reformatted the manuscript to better fit the GigaScience Technical Note format and added missing paragraphs at the end.

GigaScience has also published a number of relevant papers that may help contextualize your work. We have listed several recent examples below for your reference. Citing some of these articles—where appropriate in the Background or Discussion—would help frame your contribution within ongoing community efforts and strengthen the presentation of related tools and frameworks.
1. Howard C, Denton A, Jackson B W, et al. On the path to reference genomes for all biodiversity: laboratory protocols and lessons learned from processing over 2,000 species in the Sanger Tree of Life[J]. GigaScience, 2025, 14: giaf119.
https://urldefense.proofpoint.com/v2/url?u=https-3A__doi.org_10.1093_gigascience_giaf119&d=DwIBaQ&c=D7ByGjS34AllFgecYw0iC6Zq7qlm8uclZFI0SqQnqBo&r=fXfLn8Z4i5uP7ApDGzQr5wdkI3tCVmWIEXk31ziY2DY&m=Sa1pZETX15nvhE759BJw9ySqjT_Qr9eO3N3q-zus-MsW0uJUK54GJQ6DdRQUoLo0&s=aCZk9jZoxIU3FwoX07GkNBOj-gl021vG_pEcnMYuu8k&e=
2. Lawniczak M K N, Kocot K M, Astrin J J, et al. Best-practice guidance for Earth BioGenome Project sample collection and processing: progress and challenges in biodiverse reference genome creation[J]. GigaScience, 2025, 14: giaf041.
https://urldefense.proofpoint.com/v2/url?u=https-3A__doi.org_10.1093_gigascience_giaf041&d=DwIBaQ&c=D7ByGjS34AllFgecYw0iC6Zq7qlm8uclZFI0SqQnqBo&r=fXfLn8Z4i5uP7ApDGzQr5wdkI3tCVmWIEXk31ziY2DY&m=Sa1pZETX15nvhE759BJw9ySqjT_Qr9eO3N3q-zus-MsW0uJUK54GJQ6DdRQUoLo0&s=G00XoY13smvkFBBI0VMkI9_-c5d-0JqyQ_0miIoGVpY&e=

	- We have updated the two references now as at time of original submission they were only available as preprints.
**Additional Information:**	
**Question**	**Response**
Are you submitting this manuscript to a special series or article collection?	No
**Experimental design and statistics**<br><br>Full details of the experimental design and statistical methods used should be given in the Methods section, as detailed in our Minimum Standards Reporting Checklist. Information essential to interpreting the data presented should be made available in the figure legends.<br><br>Have you included all the information requested in your manuscript?	Yes
**Resources**<br><br>A description of all resources used, including antibodies, cell lines, animals and software tools, with enough information to allow them to be uniquely identified, should be included in the Methods section. Authors are strongly encouraged to cite Research Resource Identifiers (RRIDs) for antibodies, model organisms and tools, where possible.<br><br>Have you included the information requested as detailed in our Minimum Standards Reporting Checklist?	Yes
**Availability of data and materials**<br><br>All datasets and code on which the conclusions of the paper rely must be either included in your submission or deposited in publicly available repositories (where available and ethically appropriate), referencing such data using a unique identifier in the references and in the "Availability of Data and Materials"	No

section of your manuscript.<br><br>Have you have met the above requirement as detailed in our Minimum Standards Reporting Checklist?	
If not, please give reasons for any omissions below.<br><br>as follow-up to "**Availability of data and materials**<br><br>All datasets and code on which the conclusions of the paper rely must be either included in your submission or deposited in publicly available repositories (where available and ethically appropriate), referencing such data using a unique identifier in the references and in the "Availability of Data and Materials" section of your manuscript.<br><br>Have you have met the above requirement as detailed in our Minimum Standards Reporting Checklist?<br><br>"	At submission the data are part of a larger ENA project encompassing several unrelated RnD and production samples, and thus need to be split out to be published, but they will be made available during the review process.
GigaScience has policies and guidelines in place for the use of generative AI-writing tools such as ChatGPT. If you have used such writing tools to assist with writing the manuscript this must be declared and cited in the text. Authors should not list AI-writing tools and other AI-assisted technologies as an author or co-author and should acknowledge that they are fully responsible for text generated or refined by AI-writing tools.<p><br><br>A summary of use (particularly in the introduction or among methods) needs to be included at the end of the paper, and the outputs should also be included as a supplementary file hosted in GigaDB or other open repositories. Please read our guidelines for more information. <p><br><br>By submitting to GigaScience, you are	No

aware of the journal's AI-writing tools policy, and if you have declared use of such tools below, you have acknowledged this where appropriate in your manuscript and have made a summary of use and outputs available. <p> <b>AI-assisted writing tools have been used in the preparation of this manuscript?	

Revised manuscript Click here to access/download;Manuscript;Squish manuscript - Review.docx

Breaking the cold chain: solutions for room temperature preservation of mosquitoes leading to high quality reference genomes

Authors: Fiona Teltscher[1†], Petra Korlević[1†], Edel Sheerin[1,2], Alex Makunin[1], Mara K. N. Lawniczak[1*]

Affiliations:

1 Wellcome Sanger Institute, Hinxton, United Kingdom

2 James Cook University, Townsville, Australia

† These authors contributed equally to this project

*mara@sanger.ac.uk

Abstract

Background

The Earth BioGenome Project (EBP) is a global endeavour to produce reference genomes for all described eukaryotic species. The majority of described species are arthropods, which tend to be small and require taxonomic expertise to identify to species level. Therefore, the ability to collect and preserve specimens in a suitable way for long read and Hi-C data generation using very simple approaches with minimal infrastructure is certain to be important in scaling up reference genome generation. Field collections requiring dry ice or dry shippers can be logistically challenging to arrange, are notoriously expensive, and DNA degrades rapidly if ultra-cold temperature is not maintained, which is devastating given how expensive and time consuming field work can be.

Findings

Using *Anopheles* mosquitoes as an insect representative we evaluate how well different preservation liquids protect high molecular weight DNA, RNA, and nuclei for Hi-C at room temperature when mosquitoes are held intact versus slightly squished. We find that squished samples stored in 100% ethanol and Allprotect held at room temperature for one week result in excellent preservation of both high molecular weight DNA and nuclei for Hi-C. Other tested buffers, including RNAlater, EDTA at several pHs, and DMSO Salt Solution (DESS) performed satisfactorily for long read data generation and RNA retrieval, but less ideally for Hi-C, which may have bigger negative impacts when aiming to generate data for organisms with larger genomes.

Conclusions

We present multiple viable options for room temperature collection and/or shipment for arthropod samples, with direct suggestions for shipment and storage of mosquitoes and similarly sized specimens. Further exploration across a broader range of species will hopefully enable cheaper and more widely available reference genome generation globally.

Keywords

sample storage, sample shipment, reference genome generation, PacBio, Hi-C

Introduction

To date, only 1% of the earth's described eukaryotic species have publicly available genome data and only 0.2% of species have high quality chromosomal reference genomes (https://goat.genomehubs.org/projects/EBP). Major advances over the recent years in long read sequencing quality from Pacific Biosciences (PacBio) and Oxford Nanopore Technologies (ONT), together with demonstrated scalable extraction approaches across the tree of life [1] have highlighted the feasibility of the Earth BioGenome Project [2,3] and projects like the Darwin Tree of Life [4], which has already released genomes for over 2000 species, are putting these advances into action. On the data generation side, challenges remain in scalable assembly and curation, but perhaps the largest remaining challenge is securing sufficient funding. However, on the specimen collection side, there remain major challenges in identifying and collecting specimens at scale and collection challenges are made even more difficult by the need to collect and store with constant and reliable access to ultra cold temperatures. In practice, cold chain enabled specimen collection is managed either by collecting live specimens and returning with them to the lab where they can be rapidly processed, or through the use of dry shippers or dry ice in the field. However, all of these approaches add a logistical burden and considerable expense to field work, not to mention the risks should the cold chain method be compromised through dry ice evaporation or the dry shipper losing charge either during collection or shipping. Beyond these added expenses and risks, perhaps the greatest issue is that cold chain as a requirement precludes spontaneous collection when a species is encountered unexpectedly. Under current best practice guidelines for reference genome quality material [5], when one encounters a specimen in the field but is either unable to keep it alive until getting to the lab or cold chain access is not available, the specimen should not be collected as it will not result in the kind of quality material needed for a reference genome. This guidance would be different if there were simple preservation buffers that protect HMW DNA, RNA, and nuclei from any specimen at room temperature and this would be a game changer for scaling up species collections as theoretically, any field work

efforts could be “genome enabled”. Most arthropod biodiversity research and genome sequencing does not allow for these restrictive collection protocols, as sampling is performed far from cold storage, initial species identification can be uncertain, and would thus greatly benefit from a room temperature storage and shipment approach.

Previous work has set out to explore breaking the cold chain for vertebrate tissues [6]. This work explored ethanol, DESS, and DNAgard for several species as well as Allprotect and RNAlater for fish. A variety of temperatures and tissues were explored, and not all preservatives were tested for compatibility with either long read sequencing or Hi-C. In short, most preservatives resulted in HMW DNA preservation but ethanol and DESS preserved higher molecular weight DNA better than DNAgard, which was also poor at preserving chromatin interactions for Hi-C sequencing. Vertebrate tissue is typically processed into smaller pieces at the time of sampling for various downstream approaches. The same may not be true for many arthropods, which are often small and easily collected as whole specimens with efforts to avoid morphological damage, as morphology may be required to reach an accurate species identification. Since both DESS and ethanol have long been known as good solutions for preserving DNA, we considered that these may not penetrate the insect cuticle rapidly enough to prevent DNA degradation and several years ago, began asking collaborators on our *Anopheles* Reference Genomes project (ENA Project ID PRJEB51690) to “lightly squish” their mosquito specimens in these solutions prior to storing and shipping at room temperature to best preserve DNA [7]. Here we more systematically explored compromising the cuticle versus leaving the specimen intact in several different preservation buffers and at two different temperature regimes, either storing the specimen in the freezer before subjecting it to a period of one week at room temperature, or simply storing it for one week at room temperature. In both cases, specimens were then removed from their preservation buffers and held at -70°C until further work.

The buffers we tested here include: 1) Allprotect Tissue Reagent (Qiagen), which is optimized to protect DNA in the 10-30 kbp range, RNA, and proteins; 2) DESS or DMSO Salt Solution [8], which has been used across many species to protect DNA; 3) EDTA at three different pHs, because recent work exploring room temperature preservation of several aquatic species found that the primary protective ingredient in DESS was not the DMSO but the EDTA [9], and the same team showed that higher pH levels of EDTA can result in even greater HMW DNA recovery for some species [10]; 4) 100% ethanol, which is a common reagent used to preserve DNA at room temperature; and 5) RNA*later* (Thermo Fisher Scientific), which is a reagent that protects RNA in tissue including at room temperature. We tested each of these solutions on replicates of single *Anopheles* mosquitoes per collection tube held at room temperature for one week to ascertain which approaches result in high quality DNA, RNA, and nuclei preservation suitable for Hi-C.

Methods

Mosquito rearing and sample storage experiments

Seven to eight day old female *Anopheles coluzzii* N'Gousso strain laboratory reared mosquitoes fed on a 8% w/v fructose solution were collected with an electronic aspirator and anaesthetised using CO_2. Batches of 6 to 12 females were transferred using forceps into a glass dish containing 100% ethanol, where they remained submerged for 1-3 min, followed by dabbing each individual on a Kimwipe to remove excess ethanol and transferring into 0.5 mL DNA LoBind tubes (Eppendorf). Each tube contained 400 µl of one of the following storage solutions: 100% molecular grade ethanol 200 proof (Fisher BioReagents), DESS (**D**MSO/**E**DTA/**S**alt **S**olution, comprising DMSO 20%, EDTA 0.25 M (Fisher BioReagents, crystalline powder dissolved in distilled water), NaCl to saturation, pH adjusted to 8 with HCl 5 mol/l or NaOH 5 mol/l from VWR Chemicals), 0.25 M EDTA pH 8, 0.25 M EDTA pH 9, and 0.25 M EDTA pH 10 (same 0.25 M EDTA solution as above with further NaOH pH adjustments), and Allprotect Tissue Reagent (Qiagen, abbreviated to Allprotect throughout). As Allprotect is extremely viscous, it was not possible to ensure exactly 400 µl was in each tube, but this was approximately correct. DESS was autoclaved and all EDTA solutions were filter sterilized for longer room temperature shelf life storage.

For each storage solution two treatments were prepared in sets of five replicates: "intact" in which the mosquito was picked up by a leg or the proboscis from the petri dish and placed into the tube without any physical disruption to the carcass, and "squished" in which a mosquito was similarly placed into the tube but also lightly pressed one time against the side of the tube using a plastic pestle, typically used for grinding tissues, in order to compromise the cuticle but leave the mosquito in one piece. A detailed protocol of the collection, thorax cuticle squishing approach, shipment and post-shipment longer term storage can be found in our protocols.io SOP [7]. One set of replicates for each storage solution and treatment was stored at room temperature on a laboratory bench (20-25ºC) for one week until further processing. Samples receiving this treatment are referred to as "1wRT" (one week room temperature) throughout. A week was chosen to represent a reasonable amount of time to support collection in the field without cold chain and/or shipment to another destination without cold chain. A second set of replicates was first placed in a -20°C freezer for one week prior to being stored at room temperature for another week. Samples receiving this treatment are referred to as "ff-1wRT" (freezer first followed by one week room temperature) throughout. This design was intended to simulate a situation in which field collection had access to a -20°C freezer (e.g. live samples were brought back to a lab for processing) and to test if freezing made any substantive difference to initial preservation at room temperature. After one week

at room temperature and prior to longer term storage, mosquitoes were removed from their storage solution with clean forceps (wiped with 70% ethanol in between), transferred into new 1.5 mL DNA LoBind tubes (Eppendorf), and stored in a -70°C freezer until DNA extraction. The removal of specimens from their storage liquid enabled streamlined extractions as no thawing of buffers was required and frozen specimens could enter directly into manual grinding for DNA or RNA extraction. Five snap frozen controls were also collected by submerging CO_2 anaesthetised mosquitoes in 100% ethanol for 1-3 min, removing excess ethanol by dabbing each mosquito on a Kimwipe before placing them in 1.5 mL DNA LoBind tubes, and immediately storing in a -70°C freezer. All samples were stored at -70°C for at least one week before DNA extraction. The full experimental design is schematically represented in Figure 1A.

After assessing the best DNA preservation conditions, we selected the best performing subset of preservation liquids and also added in RNA*later* Stabilization Solution (ThermoFisher Scientific, abbreviated to RNAlater throughout) to test RNA preservation and chromatin configuration preservation for Hi-C sequencing, which are needed for new reference genome generation. One representative sample per condition was submitted for low input (LI) PacBio and Hi-C sequencing and a set of replicates for each preservation liquid was also evaluated for RNA quality. The selected storage solutions were 100% ethanol, DESS, EDTA pH 8, Allprotect, and RNAlater. For each storage solution 10 “squished” samples were prepared, five to be used for HMW DNA extraction, one for Hi-C, and four for RNA extraction. In addition, 10 snap frozen control mosquitoes were collected. We did not test the freezer first option in this second set of tests nor did we test intact mosquitoes given squished mosquitoes generally performed similarly or better. The experimental design is schematically represented in Figure 1B.

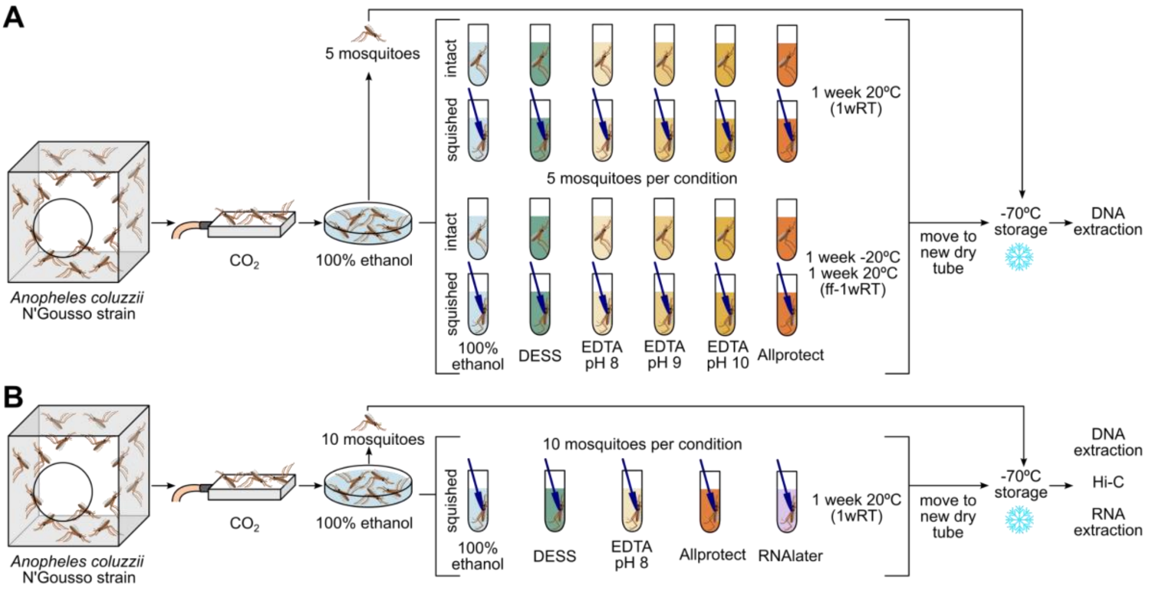

Figure 1. Schematic of our experiment simulating room temperature shipment of mosquitoes stored in various solutions. *(A) Initial experiment on a variety of storage solutions and shipping conditions. These samples were extracted but only DNA quantification and fragment size assessment was performed. (B) Repeat experiment of a subset of storage solutions, for long read and Hi-C sequencing, and RNA quality assessments.*

DNA extraction and long read PacBio sequencing

DNA was extracted using the MagAttract HMW DNA Kit (Qiagen) on the KingFisher Apex Purification System (ThermoFisher Scientific). Briefly, samples are placed on dry ice, tissue is homogenized in lysis buffer using a plastic pestle and left at 25°C for 2 h to lyse, after which the volume is transferred to a KingFisher plate and the program performs two wash steps with buffers MW1, PE and nuclease free water, with a final 400 µl elution in buffer AE [11]. Initial DNA quantification was performed using the Quant-iT PicoGreen dsDNA Assay (Invitrogen), followed by diluting each sample to about 0.25 ng/µl and re-quantifying using the Agilent Femto Pulse System using the 22 cm array and FP-1002-0275 genomic DNA 165 Kb kit following the "FP-1002-22 gDNA 165kb" method, which also assesses fragment lengths through smear analysis performed in the ProSize 4.0.0.3 data analysis software.

For the six extractions that were submitted for PacBio sequencing, the DNA was sheared to an average of 10-15 kbp using the Covaris g-TUBE system, where DNA is sheared by centrifugal force pulling it through a small orifice using a standard bench-top centrifuge. Following the Covaris manual, for each sample a total volume of 350 µl was centrifuge sheared. Due to the maximum volume of the Covaris g-TUBE being 150 µl, for each sample the volume was split into three centrifugation batches, 150+150+50 µl, and once each centrifugation was completed the batch was combined into the same 1.5 mL DNA LoBind tube. Each batch was centrifuged for a total of 6 minutes at 2,800 rpm, turning the g-TUBE every 1 min. Samples were concentrated and size selected with AMPure PB SPRI Beads (Pacific Biosciences) following the PacBio protocol "Preparing whole genome and metagenome libraries using SMRTbell prep kit 3.0" (102-166-600 April 2024) [12] with a few changes. The exact volume for each sample was roughly measured with pipette aspiration, then 3.1x 35% v/v beads were added and pipette mixed with a wide-bore tip. The sample-bead mixtures were incubated at room temperature on a tube rotor set to gentle rotation for 30 min, after which 1.5 ml of 80% v/v freshly prepared ethanol was used to wash the bead-bound DNA. DNA was eluted from the washed beads by adding 50 µl Buffer EB (Qiagen) and incubating in a benchtop shaker for 15 min at 37°C and 600 rpm.

Sheared and purified DNA was library prepped using the SMRTbell Prep Kit 3.0 (Pacific Biosciences, California, USA) as per the manufacturer's instructions. PacBio libraries for all six samples were pooled together and sequenced on one Revio SMRT Cell. Sequencing

was performed by the long read team in Scientific Operations at the Wellcome Sanger Institute.

As all of our samples performed exceptionally well with PacBio Low Input (LI) sequencing, we decided to include a previously sequenced PacBio Ultra-Low Input (ULI) sample to test the limits of chromosomal scaffolding. The sample came from the same N'Gousso colony, was collected in March of 2022, stored in 100% ethanol at room temperature for two weeks, DNA was extracted using a previously developed minimally morphologically destructive Lysis Buffer C [13,14], and purified using 2.2x AMPure XP Beads (Beckman Coulter). An aliquot containing approximately 24.3 ng of DNA was used for SMRTbell library preparation, with additional PCR amplification using the SMRTbell gDNA amplification kit (Pacific Biosciences), this PCR step being the key difference between the LI and ULI library preparation protocols. The sample was sequenced on a PacBio Sequel IIe system, again performed by Scientific Operations at the Wellcome Sanger Institute.

Hi-C sample preparation and sequencing

To evaluate whether these solutions are also suitable for preserving chromatin configuration, which is important for scaffolding reference genomes, a 1wRT squished specimen stored in each of 100% ethanol, DESS, EDTA pH 8, Allprotect, and RNAlater was submitted for Hi-C sequencing. Briefly, DNA was crosslinked and processed using the Arima-HiC v2 Kit (Arima Genomics) following the animal tissue protocol, after which Illumina libraries were sequenced on an Illumina NovaSeq X with 150 PE on the 25B flow cell aiming for >100X coverage per library.

Genome assembly

The HiFi reads were first assembled using Hifiasm 0.19.8-r603 [15] with the --primary option. Haplotypic duplications were identified and removed with purge_dups 1.2.5 [16]. The Hi-C reads were mapped to the primary contigs using bwa-mem 0.7.17 [17]. The contigs were scaffolded using the Hi-C data with YaHS 1.2.2 [18] using the --break option for handling potential mis-assemblies. The scaffolded assemblies were evaluated using PretextView 0.0.2 [19]. Contact frequencies were evaluated by digest-aware mapping of 1 M reads for each sample against contigs of AcolN3 (GCA_943734685.1) using HiLine 0.2.4 [20].

RNA extraction and quality control

DNA structure is known to be more stable than RNA, especially at room temperature, as its double stranded structure protects it from oxidation for longer, and thus we also assessed whether these preservation solutions were suitable for preserving RNA. Four 1wRT squished specimens stored in each of 100% ethanol, DESS, EDTA pH 8, Allprotect, and

RNAlater were RNA extracted using the Dynabeads mRNA DIRECT Purification Kit (Thermo Fisher Scientific) following the protocol for solid plant or animal tissue on the KingFisher Apex. Each mosquito was ground with a pestle motor in 300 µl of lysis buffer and subsequently passed through a 21G needle 3-5 times. Homogenised samples were stored on dry ice until all samples were processed, and then thawed simultaneously on wet ice and quickly spun in an Eppendorf miniSpin benchtop centrifuge (1,200 rpm for 30 sec) before transferring them to the KingFisher Apex sample plate. Magnetic Dynabeads were cleaned by placing the required volume of beads in their provided storage buffer on a magnet, removing the supernatant, adding the same volume of fresh lysis buffer, and then transferring 50 µl of washed beads into a second plate on the KingFisher Apex. The beads were then collected by the magnet, added to the lysed sample and incubated for 10 min at medium shaking speed. DNA bound beads were then washed twice with 600 µl of wash buffer A, followed by two washes with 300 µl wash buffer B. RNA was eluted in 50 µl of 10 mM Tris-HCl pH 7.5 for 3 min at 70°C and immediately placed on ice. RNA was quantified and fragment sizes explored using the Qubit RNA High Sensitivity Assay Kit (Thermo Fisher Scientific) and the High Sensitivity RNA ScreenTape Assay for TapeStation Systems (Agilent). Routine DNase treatment was done using the TURBO DNA-free Kit (Invitrogen) to remove any remaining DNA. In short, 0.1 volume of 10X TURBO DNase Buffer and 1 µl of TURBO DNase Enzyme were added to the samples in a PCR plate, gently mixed on an Eppendorf ThermoMixer at 800 rpm, then incubated at 37°C for 20 min. The DNase was then inactivated by adding 0.1 volume of DNase Inactivation Reagent, briefly shaking at 800 rpm, and incubating for 5 min at room temperature before centrifuging the plates at 2,000 g for 5 min. The supernatant was transferred to a new plate and the RNA quantity was assessed by Qubit RNA High Sensitivity Assay Kit.

Results

DNA preservation across storage solution and conditions at room temperature

We initially evaluated six storage solutions (100% ethanol, DESS, EDTA pH 8, pH 9, or pH 10, and Allprotect), two sample handling conditions (intact, squished), and two storage approaches (1wRT, ff-1wRT) for high molecular weight (HMW) DNA preservation. DNA quantity and length estimates were assessed using a Femto Pulse instrument for every replicate and condition. Due to the large number of replicates across conditions, the extractions were completed over two days, so we only tested for statistically significant patterns in intact and squished replicates for the same storage solution as they were always extracted and purified the same day using the same reagents.

For the 1wRT setup, we compared estimated total DNA amounts in ng (noting there is natural variability in mosquito size and thus DNA yields), DNA amounts for fragments above

5 kbp length, and average DNA lengths in intact versus squished samples across all storage solutions (Fig. 2A). The 5 kbp cutoff was chosen because long read sequencing approaches can use a SPRI clean-up to eliminate molecules shorter than ~5 kbp. While there seems to be a trend of slightly more DNA retrieved among squished samples in comparison to intact samples for most preservation solutions, the only significant difference in yield is found between intact and squished samples stored in DESS and EDTA pH 10, where squished mosquitoes have more DNA above 5 kbp. We also find that average DNA fragment size lengths are significantly greater among samples that are squished and preserved in DESS, EDTA pH 8, EDTA pH 9 and Allprotect in comparison to those held intact in the same solutions (Fig. 2B and Supplementary Table 1: Tab exp1).

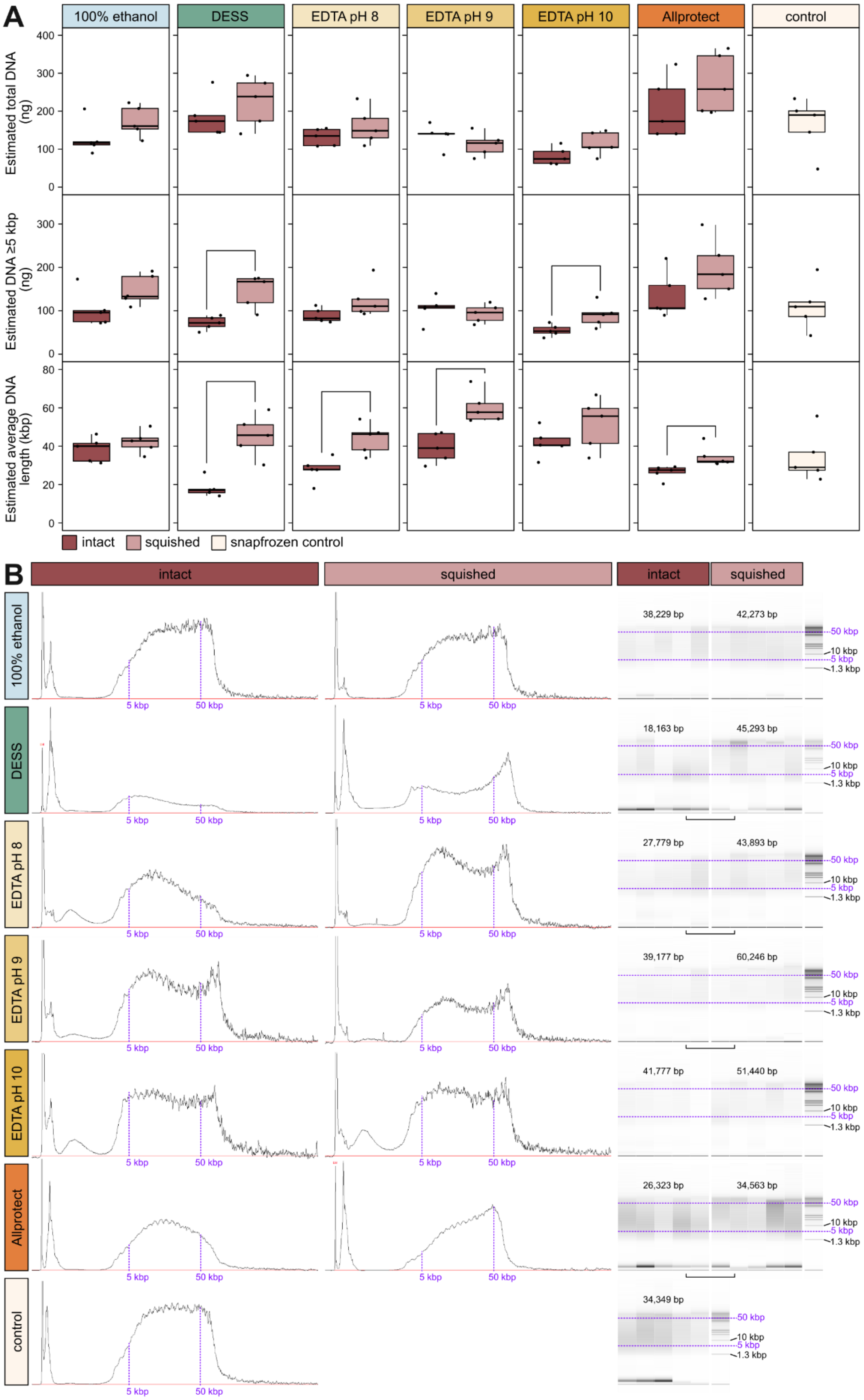
A
100% ethanol
DESS
EDTA pH 8
EDTA pH 9
EDTA pH 10
Allprotect
control
Estimated total DNA (ng)
Estimated DNA ≥5 kbp (ng)
Estimated average DNA length (kbp)
intact
squished
snapfrozen control
B
intact
squished
100% ethanol
DESS
EDTA pH 8
EDTA pH 9
EDTA pH 10
Allprotect
control
5 kbp
50 kbp
38,229 bp
42,273 bp
18,163 bp
45,293 bp
27,779 bp
43,893 bp
39,177 bp
60,246 bp
41,777 bp
51,440 bp
26,323 bp
34,563 bp
34,349 bp
10 kbp
1.3 kbp

Figure 2. DNA quantities from intact versus squished samples held at room temperature for one week. (A) *Estimated DNA yields (in nanograms) for 1wRT samples across all buffers for intact, squished, and snap frozen (control) replicates, top is total DNA, middle is only DNA above 5 kbp in length, bottom is estimated average DNA length (in kilobase pairs). Significant differences as assessed by t-test (p-value ≤0.05) are noted by brackets above (intact vs. squished).* ***(B)*** *Example Femto Pulse length profiles across buffers for a single intact (left) and squished (middle) replicate, as well as software approximations of gel smears for all replicates (right). Purple lines approximate the 5 kbp and 50 kbp lengths across samples. Average lengths across technical replicates are noted above the gel smears, with significant differences as assessed by unpaired t-tests represented by horizontal brackets. Heights are relative as each sample had a different dilution factor prior to loading (Supplementary Table 1).*

Next we evaluated the ff-1wRT samples to understand if freezing the samples at -20°C in their storage solutions would promote solution penetration and subsequently help protect more and/or longer DNA (Fig. 3A). Like 1wRT, the overall trend in ff-1wRT samples also showed higher DNA yields in squished samples compared to intact samples, with DESS, EDTA pH 8, and Allprotect solutions resulting in significantly more DNA above 5 kbp. Average fragment lengths were also significantly larger in squished samples stored in DESS, EDTA pH 8, EDTA pH 9, and Allprotect than intact samples (Fig. 3B and Supplementary Table 1: Tab exp1).

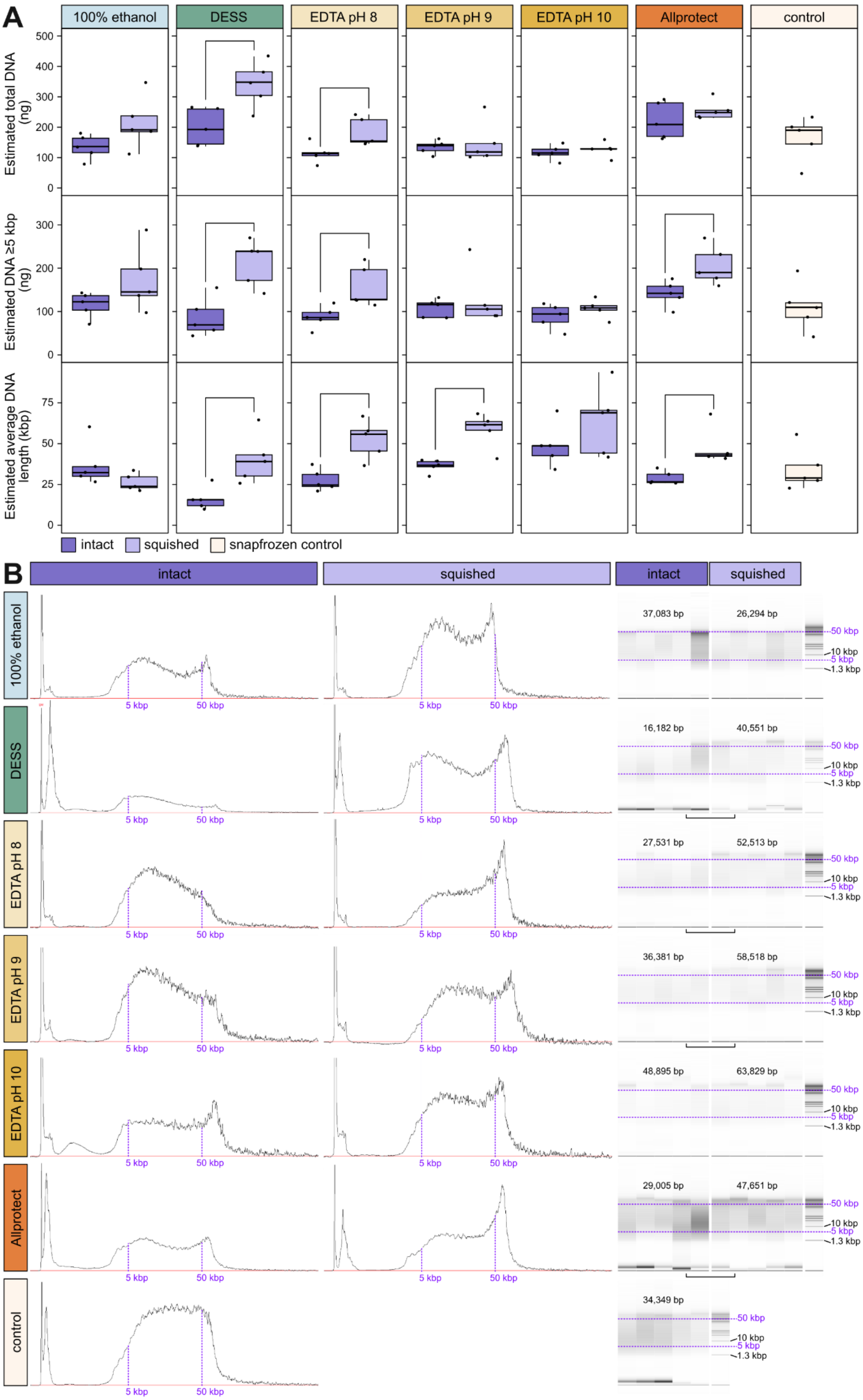
A
100% ethanol
DESS
EDTA pH 8
EDTA pH 9
EDTA pH 10
Allprotect
control
Estimated total DNA (ng)
Estimated DNA ≥5 kbp (ng)
Estimated average DNA length (kbp)
intact
squished
snapfrozen control
B
intact
squished
intact
squished
100% ethanol
DESS
EDTA pH 8
EDTA pH 9
EDTA pH 10
Allprotect
control
5 kbp
50 kbp
37,083 bp
26,294 bp
16,182 bp
40,551 bp
27,531 bp
52,513 bp
36,381 bp
58,518 bp
48,895 bp
63,829 bp
29,005 bp
47,651 bp
34,349 bp
10 kbp
1.3 kbp

Figure 3. DNA quantities from intact versus squished samples that were first frozen then held at room temperature for one week. (A) *Estimated DNA yields (ng) for ff-1wRT samples across all buffers for intact, squished, and snap frozen (control) replicates, top is total DNA and middle is only DNA above 5 kbp in length, bottom is estimated average DNA length (in kilobase pairs). Significant differences as assessed by t-test (p-value ≤0.05) are noted by brackets above (intact vs. squished).* ***(B)*** *Example Femto Pulse length profiles across buffers for a single intact (left) and squished (middle) replicate, as well as software approximations of gel smears for all replicates (right). Purple lines approximate the 5 kbp and 50 kbp lengths across samples. Average lengths across technical replicates are noted above the gel smears, with significant differences as assessed by unpaired t-tests represented by horizontal brackets. Heights are relative as each sample had a different dilution factor prior to loading (Supplementary Table 1).*

Because the squished samples across storage temperatures showed a trend towards increased DNA yields (total and above 5 kbp), as well as longer retrieved fragments, we carried out additional t-tests comparing 1wRT and ff-1wRT squished replicates across solutions. We found that ff-1wRT DESS resulted in more total DNA than 1wRT, and 100% ethanol 1wRT resulted in longer DNA fragments than ff-1wRT (Supplementary Table 1: Tab exp1 bottom).

Genome assembly outcomes across storage solutions

Current best practices in genome assembly consist of long read DNA sequencing together with Hi-C sequencing, ideally of the same specimen, to order and orient contigs. Given that all of our HMW DNA preservation conditions performed satisfactorily, with squished samples performing slightly better, we next prepared a test set to explore full genome assembly using the best performing subset of buffers including 100% ethanol, DESS, EDTA pH 8, Allprotect, and RNAlater. We selected only pH 8 among the EDTA options as it performed slightly better than the other two pHs and we also added in RNAlater for evaluation. We also selected only the 1wRT storage option because there were very minor differences between 1wRT and ff-1wRT, and the former does not require access to a -20°C freezer.

For this set of HMW DNA extractions, overall DNA yields were typically higher than for the previous set of extractions, within the normal range of variation that we observe, presumably due to differences in the size of our colony mosquitoes in any given generation. As this was a smaller sample set, we were able to extract everything in one day, and could thus compare DNA retrieval across all buffers at 1wRT and the snap frozen controls. We did not find any significant differences in the amount of DNA retrieved between the squished mosquitoes preserved at RT for one week in the solutions listed and the snap frozen controls, for total DNA and only DNA above 5kbp, which is highly promising for room temperature preservation of HMW DNA (Fig. 4A top, Supplementary Table 1: Tab exp2). Average fragment

length was also comparable amongst experimental and control samples, with only 100% ethanol samples being significantly longer than the snap frozen control samples (Fig. 4A bottom). A single representative DNA extract for each preservation solution was chosen for shearing and sequencing (Fig. 4B). Although RNAlater and Allprotect showed more degraded profiles pre-shearing, they both sheared well to around a 10 kbp size along with all of the other preservation solution and the snap frozen control extractions (9,739 bp - 11,730 bp, Fig. 4B).

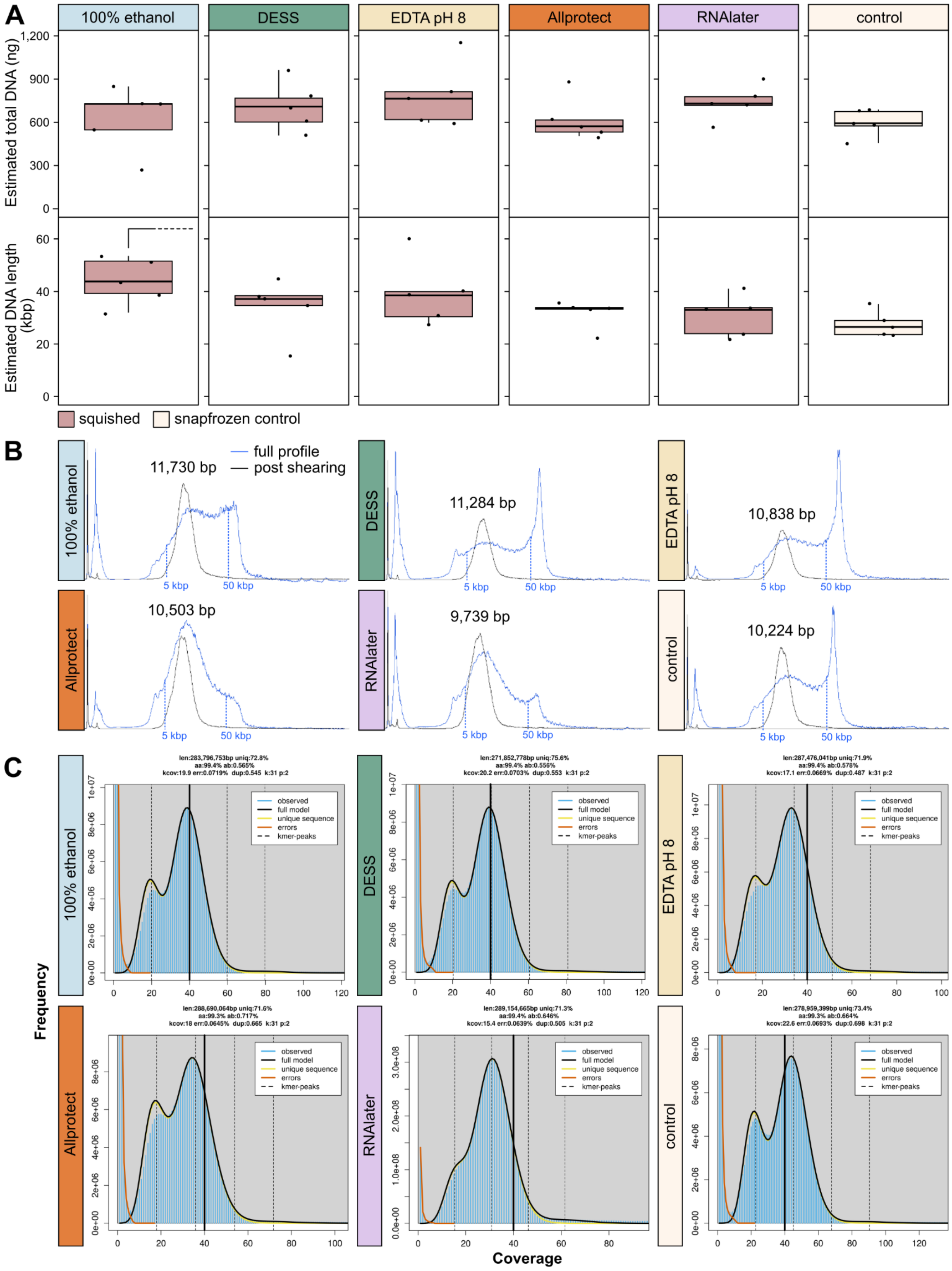

Figure 4. DNA quantities and assessments extracted from squished samples held at RT for one week. (A) *Estimated total DNA (ng) for each of the preservation solutions and snap frozen controls (top) and estimated average DNA length (kbp) (bottom). Significant differences as assessed by t-test (p-value ≤0.05) are noted by half brackets above (storage solution vs. snap frozen control).* ***(B)*** *Femto Pulse traces for the samples selected for PacBio LI sequencing for each storage solution. The blue line is the original full profile for each sample and the black line is the same sample post g-TUBE shearing. The post-shearing peak size is noted above, with vertical blue dotted lines denoting the approximate*

location of 5 kbp and 50 kbp. ***(C)*** *Resulting k-mer profiles for each sequenced PacBio library. Summary text above each plot summarizes haploid genome length (len), % unique sequences (uniq), percent homozygous k-mers (aa), percent heterozygous k-mers (ab), inferred coverage (kcov), read error rate (err), fraction duplicated reads (dup), k-mer size (k), ploidy (p). The colored lines showcase observed k-mer coverage frequencies (blue), the fitted model for all (black), unique (yellow), and error (red) k-mers. The thicker black line denotes a 40x coverage.*

Each of the six sheared DNAs had PacBio libraries prepared and these were sequenced on a single Revio cell, aiming for > 15x coverage per haplotype per specimen. The *Anopheles coluzzii* genome size is approximately 250 Mbp, which led to this plexing decision. We achieved excellent sequencing results for each of the six libraries, with a typical N50 fragment length of > 10 kbp (Table 1) and GenomeScope profiles that suggested a successful sequencing run (Fig. 4C). This indicates that none of the preservation buffers caused DNA damage that would have resulted in reduced yields.

Table 1. *PacBio sequencing and contig assembly summary for six samples sequenced using LI and one using ULI.*

**Storage solution**	**k-cov**	**Haploid size**	**Repeat (%)**	**Het. (%)**	**yield**	**N50**	**Contig length**	**Contig number**	**Contig N50 (L50)**
100% ethanol	19.91	283,796,753	27.29	0.57	11,591,002,911	11,777	270,585,947	135	16,642,641 (5)
DESS	20.20	271,852,778	24.48	0.56	11,261,944,002	10,891	271,115,124	174	15,936,013 (6)
EDTA pH 8	17.07	287,476,041	28.17	0.59	10,048,123,268	11,208	272,880,309	183	17,949,691 (6)
Allprotect	17.95	288,690,064	28.47	0.73	10,610,020,181	10,452	273,620,267	165	14,159,000 (6)
RNAlater	15.40	289,154,665	28.78	0.67	9,112,472,994	10,219	272,936,586	163	14,505,387 (6)
Control	22.59	278,959,399	26.70	0.67	12,913,361,369	11,455	278,482,832	127	16,790,217 (6)
ULI	19.49	248,552,781	21.21	0.74	10,109,927,058	8,187	262,372,469	846	1,003,577 (80)

Due to the small size of the mosquitoes and the need to use an entire mosquito's DNA extract for long read sequencing, for each preservation solution, Hi-C data was generated from a second individual. Mapping of Hi-C reads to reference contigs demonstrates lower

frequency of long-range contacts in DESS, EDTA pH8 and RNAlater, which is likely to negatively impact scaffolding in repeat-rich genomic regions (Figure 5). We evaluated the performance of Hi-C data generated from squished specimens stored in each of the different preservation solutions for efficient chromosome level scaffolding using two approaches. First, we self scaffolded, meaning we used the long read data from the same preservation approach as was used for the Hi-C data (e.g. the two squished individuals that were both stored in 100% ethanol and used for long read and Hi-C sequencing). However, self scaffolding results were not particularly informative due to the high quality of the PacBio LI data for each specimen. Reconstruction of the genome for every preservation test resulted in reconstruction of scaffolds corresponding to three expected chromosomes 2, 3, and X (control, 100% ethanol, Allprotect) or excessively merging chromosomes over telomeres (DESS, RNAlater), a common issue that can be routinely fixed in curation. The only exception was EDTA, where six largest scaffolds correspond to five chromosome arms (2R, 2L, 3R, 3L, X), indicating that at least one chromosome arm could not be reconstructed. This was largely due to >1 Mbp contigs for >90% of the assembly from the long read data alone, so even a relatively low Hi-C signal was sufficient to merge these large contigs together (Table 2, Figure 6A).

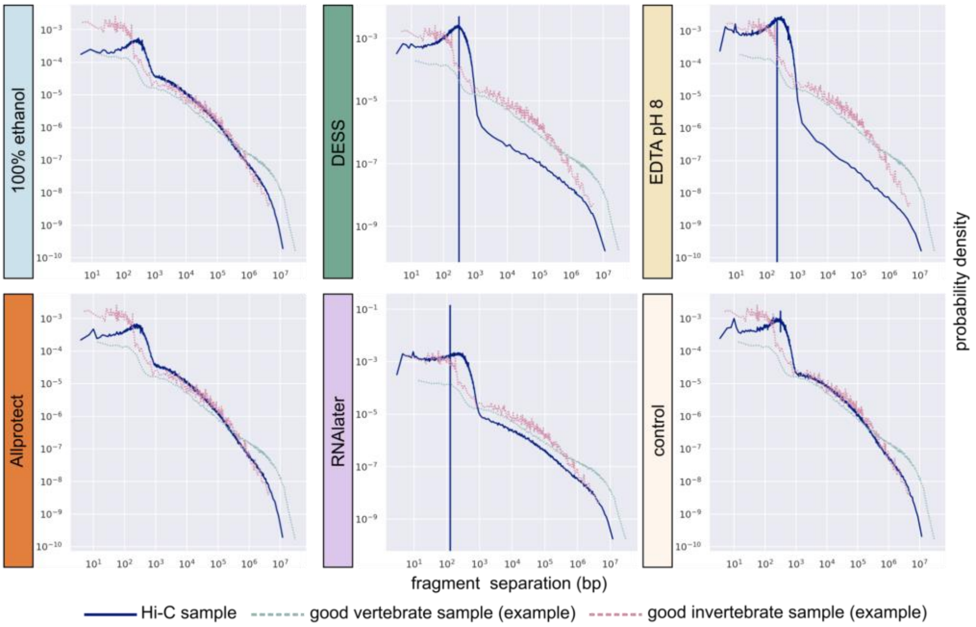

Figure 5. Fragment separation frequencies for Hi-C sequencing runs. *The dark blue lines are actual observations for samples stored across our tested solutions, the green dotted lines show the theoretical distribution of a good vertebrate example and the pink dotted lines show the theoretical distribution of a good invertebrate example (same two theoretical lines in all six subfigures). The probability density on the y axis shows proportion of chromosomal contacts for a given contact separation, and the x axis shows the fragment separation in bp.*

Table 2. *Hi-C scaffolding summary to newly sequenced PacBio Low Input (LI) samples and one previously sequenced PacBio Ultra-Low Input (ULI) sample. For "self scaffolding", we used the PacBio LI library and Hi-C library from different individuals preserved using the same storage solutions. For the "ULI scaffolding" approach, the same ULI sample was scaffolded using Hi-C data from each of the different storage solutions.*

**Storage solution**	**Hi-C coverage**	**Self scaffolding**			**ULI scaffolding**		
		**Scaffold number**	**Scaffold N80 (L80)**	**Scaffold N90 (L90)**	**Scaffold number**	**Scaffold N80 (L80)**	**Scaffold N90 (L90)**
100% ethanol	504.1	112	2,927,161 (5)	2,352,000 (6)	316	2,556,046 (5)	791,104 (15)
DESS	377.5	150	4,135,150 (4)	2,208,000 (5)	561	3,625,167 (5)	314,510 (41)
EDTA pH 8	367.0	155	19,127,350 (6)	5,573,949 (8)	694	4,251,698 (11)	281,865 (45)
Allprotect	447.0	140	3,228,000 (5)	3,030,157 (6)	348	2,168,088 (5)	484,000 (20)
RNAlater	502.8	136	3,358,947 (4)	2,806,863 (5)	441	2,411,669 (4)	316,772 (37)
Control	517.2	104	4,903,000 (5)	4,514,151 (6)	328	2,402,669 (5)	645,855 (17)

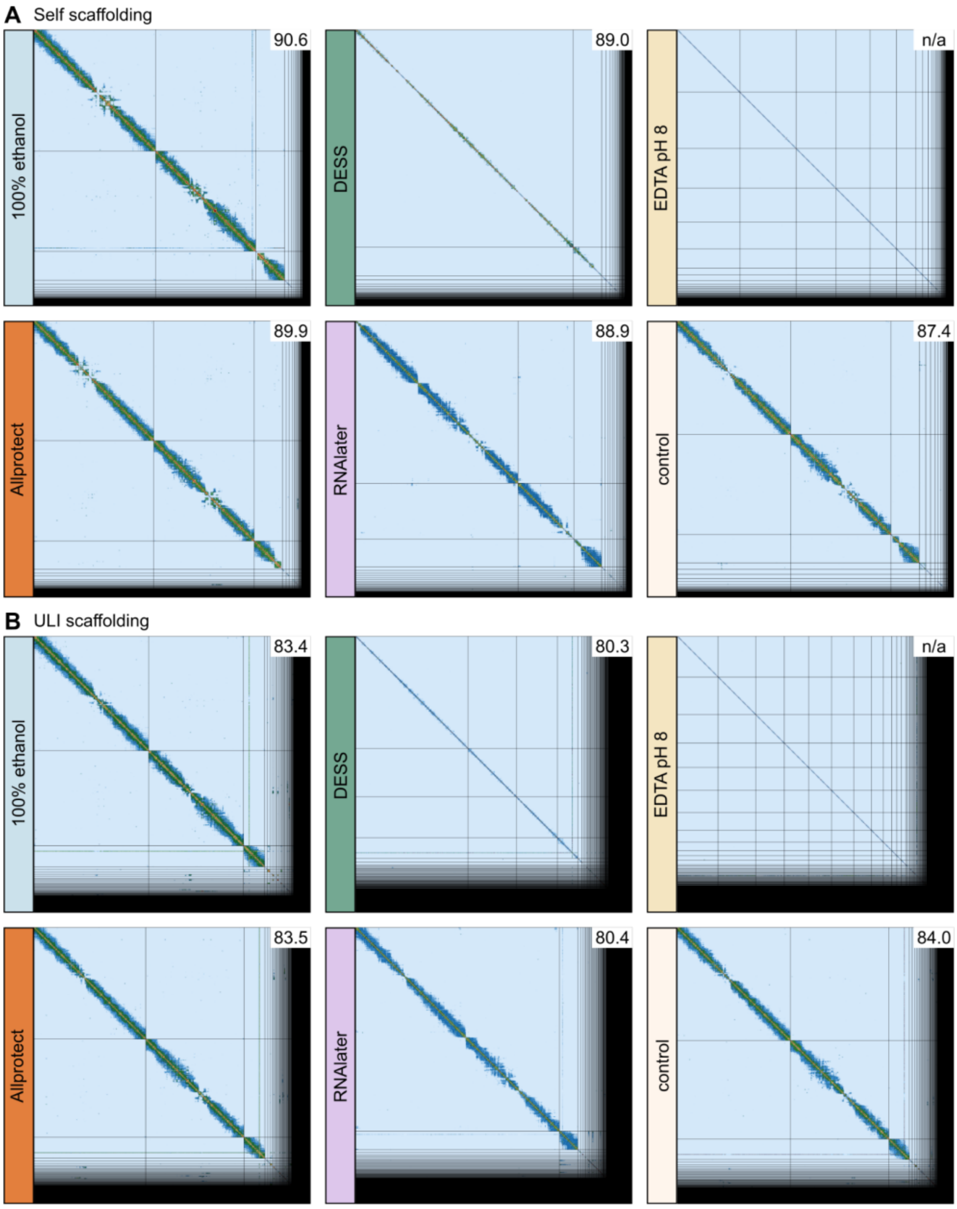

Figure 6. Scaffolded Hi-C plot for different storage solutions. *(A) Self scaffolding. (B) ULI sample scaffolding. The grid for each preservation approach corresponds to the resulting scaffolds in decreasing size order, the color corresponds to the Hi-C contact frequencies - further from diagonal are more long-range contacts. The numbers in the upper right corner of each Hi-C plot showcase the percentage of assembly that fall into chromosomal scaffolds, highlighting how much is leftover in the small scaffolds at the right*

Second, we used each of the Hi-C datasets to scaffold a much lower contiguity long read assembly derived from a previously sequenced specimen from the same mosquito colony that was sequenced using PacBio Ultra Low Input (ULI) (Table 1 last row). PacBio ULI uses a PCR amplification step to overcome low quantities of DNA, and has recently been replaced by PacBio AmpliFi. The ULI contig assembly has lower N50 fragment lengths (8,187 kbp) and the L50 (number of contigs in which 50% of the genome is covered) is at 80, whereas for all

six LI samples, the L50 was 5 or 6. This much more fragmented ULI contig assembly provides a better assessment of the scaffolding success of each of the Hi-C libraries, as the contig assembly is in over 800 pieces as opposed to under 200, therefore the scaffolding outcome depends much more on long-range contact information from the Hi-C data. In this ULI scaffolding analysis, the most comparable conditions to the snap frozen control were 100% ethanol and Allprotect, with similar Scaffold N90 and L90 values (Table 2). DESS and EDTA pH 8 showed both decreasing long-range contact frequency (Fig. 6B) and decreasing efficiency in scaffolding the ULI sample when comparing the N80 (L80) and N90 (L90) scaffolds (in which we showcase how many scaffolds fit 80% and 90% of the genome, respectively, and note a larger jump in L90 for those preservation solutions) (Table 2). RNAlater was inconsistent, performing similarly to DESS and EDTA for scaffolding (Table 2), yet showing more long range contacts than these preservatives (Figure 6).

Finally, we also examined the quantity and quality of RNA retrieved from each of the preservation solutions. mRNA quantity was evaluated before and after DNase treatment, with a t-test comparing conditions to the snap frozen control finding that the average RNA amounts were significantly lower in 100% ethanol and Allprotect, but all preservatives resulted in similar average RNA fragment lengths (Supplementary Table 1: Tab exp2 bottom, Fig. 7). Evaluating RNA profile quality is difficult as insects do not reliably have the distinct rRNA bands that permit the use of the RNA Integrity Number (RIN), thus we inspected the profiles for visible signs of degradation. In comparison to snap frozen control, there are signs that DESS and EDTA pH 8 one week room temperature preserved samples show more degraded RNA (Fig. 7B). In contrast, RNAlater and Allprotect, which are marketed as RNA preservation solutions by their respective providers, have RNA profiles similar to the snap-frozen controls (Fig. 7B).

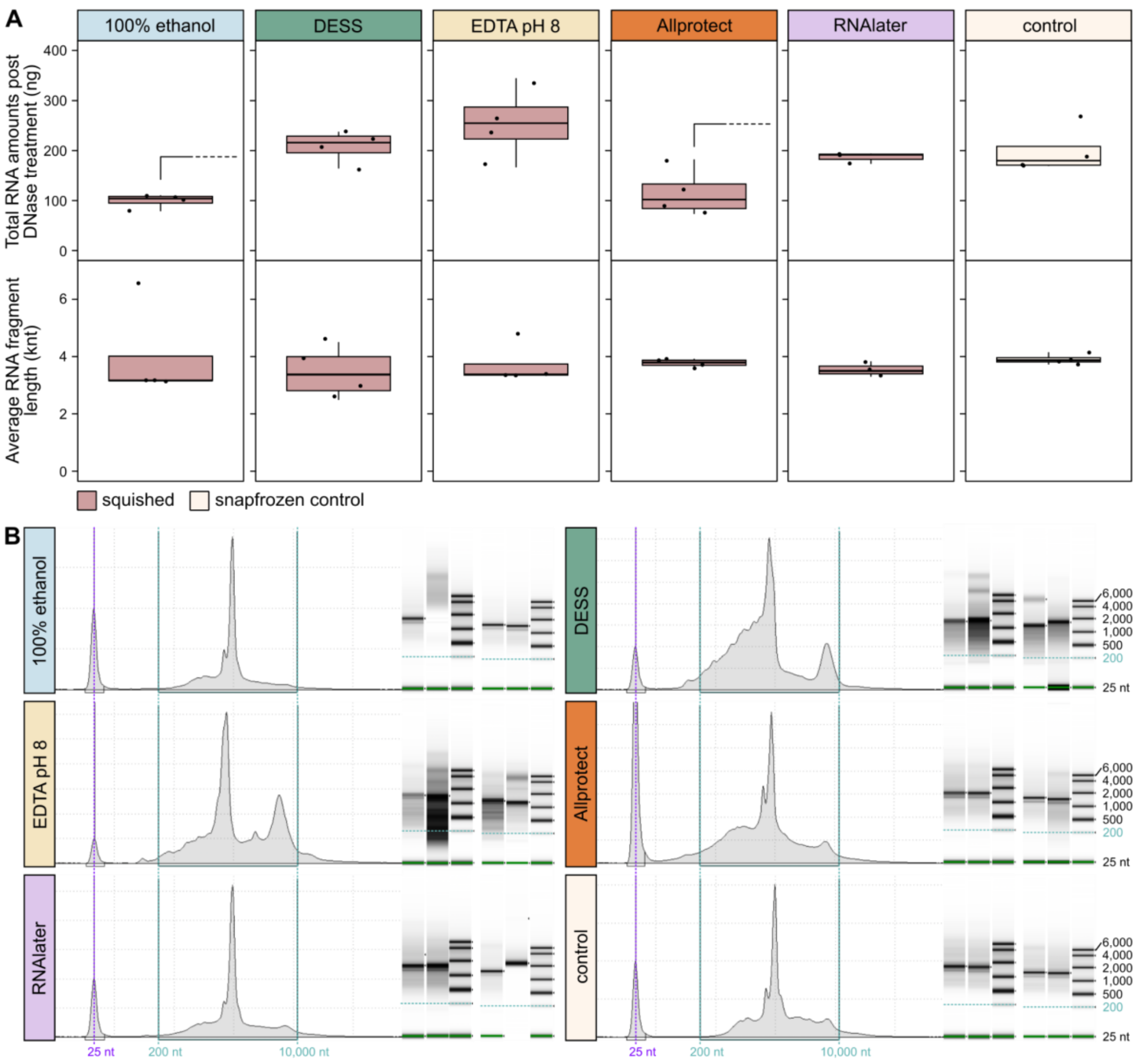

Figure 7. RNA retrieval from different storage solutions. *(A) Four squished 1wRT samples per preservation solution with quantities measured with the qubit HS RNA kit after DNAse treatment (top) and average RNA length measured with the High Sensitivity RNA ScreenTape Assay for TapeStation Systems. Significant differences as assessed by t-test (p-value ≤0.05) are noted by half brackets above (storage solution vs. snafprozen control). (B) Example RNA profiles generated on the TapeStation. For each preservation solution, two replicates were run separately and gel representations of each of the four profiles together with their ladders can be seen to the right in each subplot. Purple vertical lines denote the 25 nt marker, with two vertical green lines denoting the 200 nt and 10,000 nt range, with the 200 nt line also being noted horizontally across the gel representations.*

Discussion

Snap freezing tissue and/or specimens and retaining them at ultra cold temperatures is considered best practice for long read, Hi-C, and RNAseq data generation, but snap freezing in the field is logistically challenging and expensive and requires preparatory work to ensure adequate access to dry shippers or dry ice. Even when collecting using ultra cold storage is possible, the material may need to be shipped elsewhere for data production, and shipping

with dry ice or in dry shippers results in considerable expense as well as risk of cold chain loss. In our experience, shipping mosquito samples on dry ice from Cameroon to the UK would have been considerably more expensive (several thousand GBP) than the production costs of creating the reference genome for the species. As a result of cold-chain costs and risks, we have been trialling different room temperature preservation approaches to support cold chain free collection and shipping for insects. These trials led to the development of the squish method [7] and also successful genome generation for the samples that were eventually shipped to the UK at room temperature from Cameroon [21]. However, we did not systematically compare different preservation approaches in a controlled fashion. Here, using colony mosquitoes reared in the UK, we evaluated a number of preservation solutions and storage conditions to more thoroughly explore what short-term cold chain free preservation approaches can lead to high quality reference genomes for insects.

We find that all preservation solutions and storage conditions tested here preserve sufficient HMW DNA for *Anopheles* mosquitoes held at room temperature for one week, and long read data generated from squished mosquitoes for each of the five different preservation solutions tested here was indistinguishable from long read data from a snap frozen control. The quantity of HMW DNA that can be extracted from a specimen is dependent on the size of the tissue/specimen, and the quantity of DNA that is needed is dependent on the genome size of the organism. Thus, while the specific preservation solutions used for these small mosquitoes did not matter for the quantity and quality of the resulting long read data, for larger genome organisms or smaller body sizes these small differences in quantity and quality may indeed matter. For most preservation solutions, when we explore molecules above 5 kbp, we see that squishing insects results in better preservation of longer molecules than storing insects intact, suggesting that squishing may enable more rapid penetration of all solutions at room temperature. More generally, we find that squishing mosquitoes in their preservation solution prior to room temperature storage for one week tends to preserve a bit more and longer HMW DNA. We also find that there does not seem to be a major impact of freezing tissues at -20°C prior to exposing them to a period of time at room temperature, but generally advise that best results are likely to be obtained by keeping specimens at the coldest possible temperatures at all times.

For a number of promising preservation solutions, we also evaluated the quality of Hi-C data for samples squished in these solutions. We find that squished samples in 100% ethanol and Allprotect performed comparably to the snap frozen control with respect to their ability to scaffold a relatively fragmented long read ULI contig assembly. The other buffers (RNAlater, DESS, EDTA pH 8) also achieved some scaffolding but generally performed less well. Of note is that we had >100x coverage of Hi-C data for each sample, if projects were to generate much less Hi-C data or begin with more fragmented contig assemblies or larger

genomes, these latter buffers may perform much worse. In summary, RNAlater, DESS, and EDTA pH8 are likely not preserving insect chromatin conformation as well as 100% ethanol and Allprotect, and as a result, these latter two preservation buffers should be prioritised for room temperature storage if Hi-C is a necessary data type, especially if the target organism has a large genome.

RNA yields were similar across different preservation approaches, albeit slightly lower in 100% ethanol and Allprotect, and while quality is somewhat difficult to evaluate in insects, the buffers marketed for RNA preservation (Allprotect, RNAlater) were the most consistently similar to snap frozen controls. It is not widely appreciated but > 96% ethanol does preserve arthropod RNA although generally not as well as other solutions, especially for longer (> 1 week) periods at room temperature [22–24]. The results across all solutions with their performance ratings are summarized in Table 3.

Table 3. *Storage solution summary across PacBio LI sequencing, Hi-C sequencing and RNA retrieval. For RNA we only designate them as "good" or "average" because we have not sequenced the samples and are just relying on raw QC data.*

**Solution**	**PacBio LI**	**Hi-C**	**RNA**	**Final rating**
**100% ethanol**	excellent	excellent	good	PacBio, Hi-C
**DESS**	good	good	average	good
**EDTA pH 8**	poor	poor	average	poor
**Allprotect**	excellent	excellent	good	PacBio, Hi-C, RNA
**RNAlater**	good	good	good	RNA
**Control**	excellent	excellent	good	PacBio, Hi-C, RNA

Given the results above, we recommend squishing arthropods lightly in either 100% ethanol or Allprotect to be the best "one size fits all" preservation buffers for short term (< 1 week) room temperature storage with a view to using such preserved specimens to create reference genomes. Unfortunately, Allprotect is expensive at approximately $10/mL (Qiagen website), and as it is extremely viscous, it is nearly impossible to dispense accurate volumes using the pump nozzle bottle it is supplied in. An alternative approach if cost is a consideration and if tissues are not in short supply is to split a specimen into two parts – one part preserved in 100% ethanol for DNA and Hi-C and one part (or some specific tissues of interest) into RNAlater for RNA sequencing, as RNAlater is about 10-fold cheaper than Allprotect. Additionally, if Hi-C is not needed, or the ambition is to sequence ultra long molecules, e.g.

using Oxford Nanopore Technologies, then EDTA-based preservation approaches may be more appropriate as they tend to preserve longer molecules (Fig. 4). This requires further testing to ensure sequencing compatibility of EDTA preserved specimens with ONT. Other recent work evaluated the performance of ethanol and DESS as a tissue preservation buffer across ten species during thawing prior to HMW DNA extraction and found EDTA resulted in better protection of HMW DNA [25], however, Hi-C success was not evaluated in this work.

Benefits of squishing insects in their preservation solution is likely to extend to other arthropods outside Diptera, but further testing would be beneficial. We acknowledge the limitations in applying our approach on other arthropods, especially those with thicker cuticles (such as beetles) or living in water environments (such as crustaceans), and further work should be done to make sure the same approach, with potential slight modifications, works across different taxa. For all preservation buffers, the tissue size-to-preservation buffer volume ratios are important – here we opted for 400 µl of buffer for single mosquitoes, which typically weigh about 3-5 mg. It is also important to note the tube size should be adequate to squish the tissues and fill the tube so the sample does not risk becoming stuck in the lid out of the preservation liquid during the shipping or storage periods. Here, we also removed specimens from their preservation liquid after the room temperature storage period and before longer term freezing at -70°C. In any case, people seeking to use preservation buffers may wish to consider whether removal from the preservation buffer is required or whether this can be done at the time of further work taking place. One advantage to holding tissues long term in their preservation liquid is if the long term storage freezer fails, the tissue is likely to still be protected.

In short, factors like tissue to buffer volume ratio, tissue type, and longer term ultra cold storage approaches should all be considered when preparing to collect and ship specimens of different species. Chordates and large invertebrates are typically dissected into different tissues and/or into small pieces to fit into storage tubes, and this may improve tissue penetration of preservation solutions, as well as make it possible to obtain HMW DNA, Hi-C and RNA from a single individual. Users should factor in their own circumstances to determine which approach is best for their particular needs. For example, if the species is relatively small and/or has a large genome, the amount of HMW DNA quantity that can be extracted might be near the boundary of what is needed for long read sequencing without amplification. Thus, even though they may not be statistically significant, trends towards small increases in yield observed here with certain buffers and conditions may be beneficial. Difficult to find species may result in only one specimen collected, in which case it may merit using Allprotect regardless of its cost. In other cases, for example if collecting very large numbers of specimens, preservation solution cost may be an important factor, in which case a combination of 100% ethanol and RNAlater might be the best option. To meet the grand ambitions of the

Earth BioGenome Project, there is a major need to reduce costs associated with collecting and shipping specimens, including the high costs and inconveniences of cold chain collections and shipping. Here we present results supporting that short term room temperature storage in a wide range of buffers can lead to adequate preservation of insect material for reference genome creation. This is of special interest for dark taxa, as large sets of unknown species can be collected at a relatively low price, before they can be identified down to species level and further sent for relevant sequencing once back in a laboratory setting. There may be merits in efforts explicitly collecting for reference genomes to use these preservation buffers even if cold chain access is available given the protection to the specimens these buffers afford together with the quality of the resulting data. Future work is needed to expand tests on different preservation approaches to other taxa and perhaps also to develop lower cost buffers that maximally future proof specimens not only for RNA, DNA, and nuclei but also for morphology, proteins, and metabolites, none of which were evaluated here but all of which are of interest.

Data Availability

Raw sequencing data are available in ENA study PRJEB98990. Processed data (assembly genomics fasta files, BUSCO result, Hi-C analysis data) are available in the GigaScience repository, GigaDB [REF].

Additional Files

Supplementary Table S1. DNA and RNA QC values across all replicates. **Tab 1:** DNA quality control and relevant t-tests across conditions for six room temperature storage solutions (100% Ethanol, DESS, EDTA pH 8, EDTA pH 9, EDTA pH 10, Allprotect). **Tab 2:** DNA and RNA quality controls of five room temperature storage solutions (100% Ethanol, DESS, EDTA pH 8, Allprotect, RNAlater). In both tabs statistically significant p-values are noted in italic and red.

List of abbreviations

1wRT: 1 Week at Room Temperature; DESS: DMSO EDTA Salt Solution; DMSO: Dimethyl Sulfoxide; EBP: Earth BioGenome Project; EDTA: Ethylenediaminetetraacetic Acid; ENA: European Nucleotide Archive; ff-1wRT: Freezer First; 1 Week at Room Temperature; GBP: British Pound Sterling; HMW: High Molecular Weight; knt: kilonucleotides; LI: Low Input; ONT: Oxford Nanopore Technologies; PacBio: Pacific Biosciences; QC: Quality Control; RIN: RNA Integrity Number; ULI: Ultra-Low Input

Ethics approval and consent to participate

Not applicable.

Consent for publication

Not applicable.

Competing interests

The authors declare that they have no competing interests.

Funding

All authors and the work contained within were funded by Wellcome award 220540/Z/20/A to the Wellcome Sanger Institute. The Bill & Melinda Gates Foundation Award INV-009760 to MKNL to build high quality reference genomes from wild caught *Anopheles* mosquitoes was also instrumental in motivating the work.

Author's contributions

M.K.N.L., F.T. and P.K. contributed to the design of this paper. F.T and E.S. did all the laboratory work. A.M. ran all the bioinformatics tools for final QC and scaffolding metrics. F.T. and P.K. analysed the final data and prepared all plots and tables. M.K.N.L., F.T. and P.K. wrote the paper with inputs from E.S. and A.M.

Acknowledgements

We thank Katharina von Wyschetzki for preliminary work to evaluate several preservation approaches discussed here. We thank Cameron Robert Ferguson for the ULI sample. We thank Sanger Scientific Operations for completing all long read and Hi-C sequencing. We thank the Tree of Life Assembly team for automated genomic data processing, which includes QC and initial contig assembly.

References

1. Howard C, Denton A, Jackson BW, Bates A, Jay J, Yatsenko H, et al.. On the path to reference genomes for all biodiversity: laboratory protocols and lessons learned from processing over 2,000 species in the Sanger Tree of Life. *Gigascience*. Oxford University Press (OUP); 14:giaf1192025;

2. Lewin HA, Robinson GE, Kress WJ, Baker WJ, Coddington J, Crandall KA, et al.. Earth

BioGenome Project: Sequencing life for the future of life. *Proc Natl Acad Sci U S A*. Proceedings of the National Academy of Sciences; 115:4325–332018;

3. Lewin HA, Richards S, Lieberman Aiden E, Allende ML, Archibald JM, Bálint M, et al.. The Earth BioGenome Project 2020: Starting the clock. *Proc Natl Acad Sci U S A*. Proceedings of the National Academy of Sciences; 119:e21156351182022;

4. Darwin Tree of Life Project Consortium. Sequence locally, think globally: The Darwin Tree of Life Project. *Proc Natl Acad Sci U S A*. Proceedings of the National Academy of Sciences; 119:e21156421182022;

5. Lawniczak MKN, Kocot KM, Astrin JJ, Blaxter M, Sotero-Caio CG, Barker KB, et al.. Best-practice guidance for Earth BioGenome Project sample collection and processing: progress and challenges in biodiverse reference genome creation. *Gigascience*. Oxford Academic; 14:Not Available2025;

6. Dahn HA, Mountcastle J, Balacco J, Winkler S, Bista I, Schmitt AD, et al.. Benchmarking ultra-high molecular weight DNA preservation methods for long-read and long-range sequencing. *Gigascience*. Oxford University Press (OUP); 11:giac0682022;

7. Teltscher F, Lawniczak M: Squishing insects for preservation of HMW DNA in the field. protocols.io. https://www.protocols.io/view/squishing-insects-for-preservation-of-hmw-dna-in-t-4r3l2224jl1y/v1 (2023). Accessed 2026 May 7.

8. Seutin G, White BN, Boag PT. Preservation of avian blood and tissue samples for DNA analyses. *Can J Zool*. Canadian Science Publishing; 69:82–901991;

9. Sharpe A, Barrios S, Gayer S, Allan-Perkins E, Stein D, Appiah-Madson HJ, et al.. DESS deconstructed: Is EDTA solely responsible for protection of high molecular weight DNA in this common tissue preservative? *PLoS One*. Public Library of Science (PLoS); 15:e02373562020;

10. DeSanctis ML, Soranno EA, Messner E, Wang Z, Turner EM, Falco R, et al.. Greater than pH 8: The pH dependence of EDTA as a preservative of high molecular weight DNA in biological samples. *PLoS One*. 18:e02808072023;

11. Teltscher F, Lawniczak M. Automated High Throughput Qiagen MagAttract High Molecular Weight DNA Extraction from Mosquitoes. 2025;

12. PacBio. Preparing whole genome and metagenome libraries using SMRTbell® prep kit 3.0.

13. Korlević P, McAlister E, Mayho M, Makunin A, Flicek P, Lawniczak MKN. A minimally morphologically destructive approach for DNA retrieval and whole-genome shotgun sequencing of pinned historic dipteran vector species. *Genome Biol Evol*. Oxford University Press (OUP); 2021; doi: 10.1093/gbe/evab226.

14. Korlević P, Lawniczak M: SOP - Lysis C plate based DNA extraction. protocols.io. https://www.protocols.io/view/sop-lysis-c-plate-based-dna-extraction-ewov1o3molr2/v1

(2023). Accessed 2026 May 11.

15. Cheng H, Concepcion GT, Feng X, Zhang H, Li H. Haplotype-resolved de novo assembly using phased assembly graphs with hifiasm. *Nat Methods*. Springer Science and Business Media LLC; 18:170–52021;

16. Guan D, McCarthy SA, Wood J, Howe K, Wang Y, Durbin R. Identifying and removing haplotypic duplication in primary genome assemblies. *Bioinformatics*. Oxford University Press (OUP); 36:2896–82020;

17. Li H. Aligning sequence reads, clone sequences and assembly contigs with BWA-MEM. arXiv [q-bio.GN].

18. Zhou C, McCarthy SA, Durbin R. YaHS: yet another Hi-C scaffolding tool. *Bioinformatics*. Oxford University Press (OUP); 39:btac8082023;

19. Harry E, Guan S. PretextView: OpenGL Powered Pretext Contact Map Viewer. Github;

20. . HiLine: HiC alignment and classification pipeline. Github;

21. Nsango SN, Agbor J-P, Ayala D, Johnson HF, Heaton H, Wagah MG, et al.. A chromosomal reference genome sequence for the malaria mosquito, Anopheles moucheti, Evans, 1925. *Wellcome Open Res*. Wellcome Open Res; 8:5072023;

22. Torres MG, Weakley AM, Hibbert JD, Kirstein OD, Lanzaro GC, Lee Y. Ethanol as a potential mosquito sample storage medium for RNA preservation. *F1000Res*. F1000Res; 8:14312019;

23. Hasegawa N, Techer M, Mikheyev AS. A toolkit for studying Varroa genomics and transcriptomics: preservation, extraction, and sequencing library preparation. *BMC Genomics*. Springer Science and Business Media LLC; 22:542021;

24. Kono N, Nakamura H, Ito Y, Tomita M, Arakawa K. Evaluation of the impact of RNA preservation methods of spiders for de novo transcriptome assembly. *Mol Ecol Resour*. Wiley; 16:662–722016;

25. Messner E, Becker L, DeSanctis ML, Soranno EA, Pianka R, Pierce C, et al.. Perish the thawed? EDTA reduces DNA degradation during extraction from frozen tissue. *PLoS One*. Public Library of Science; 20:e03218722025;

We thank the reviewers and editor for their insightful comments, and have included most of their suggestions into the manuscript. For the suggestions that require additional labwork we have hopefully made it clear why that is out of the scope of this paper. All new sentences and paragraphs are highlighted with red text in the resubmission. The formatting has also been updated to include several paragraphs that are mandatory in a GigaScience Technical Note publication. Below we go through each comment separately.

Reviewer 1:

This study uses *Anopheles coluzzii* as a model to systematically evaluate preservation methods for generating high-quality genomic and Hi-C data, addressing a question of clear practical relevance. The development of approaches that eliminate the need for cold-chain transport is a particularly important step forward for field-based genomics, where maintaining low temperatures is often logistically difficult. In this context, the work provides useful experimental evidence and practical guidance with broad potential value. That said, several aspects of the manuscript would benefit from further clarification and refinement, as outlined below：

1. The study is conducted exclusively on *Anopheles coluzzii* under controlled laboratory conditions. While the results are promising, the extent to which these preservation strategies can be applied to other arthropods remains unclear. Given the substantial diversity in cuticle structure, body size, and physiological properties across taxa, the authors should more explicitly discuss the limitations of extrapolating their findings. Inclusion of additional taxa would be ideal; alternatively, a more thorough discussion of potential constraints would improve the manuscript.

We have now added a small paragraph explaining the limitations of the result interpretation when applying these methods to any other arthropod species. As a lab focused on mosquitoes with an in house An. coluzzii colony, it was the easiest and most minimally variable organism we could use across all tests to minimize internal sample bias. We have since sequenced several reference genomes using the above approach for Anopheles samples that were sent squished at room temperature, they are still going through curation but will be published soon as genome notes.

2. The evaluation of Hi-C data quality is primarily based on scaffolding performance, including analyses using both high-quality and fragmented assemblies. However, the Hi-C datasets were generated at very high sequencing depth (>100×), which may obscure differences among preservation treatments by compensating for variations in data quality. The authors should clarify how they distinguish genuine preservation effects from depth-related

compensation. Additional analyses based on downsampled Hi-C datasets would provide a more realistic assessment of performance under typical sequencing conditions.

Hi-C sequencing was indeed done at very high coverage to ensure saturation with long-range interactions. In order to assess Hi-C quality, we have added plots of fragment separation frequencies in log-scale (new figure, now named Fig. 5). These plots highlight better performance for ethanol and AllProtect treated samples, reduction of long-range interaction capture in RNAlater, and dramatic underperforming DESS and EDTA. Note that these patterns are also reflected in scaffolding performance in most complex 10-20% of mosquito genomes representing repeat rich heterochromatin (black bars in Fig. 6).

3. I am particularly interested in the chromosome anchoring rates observed in the Hi-C scaffolding analyses. It would be valuable for the authors to clarify whether Hi-C data generated from different preservation methods lead to differences in chromosome anchoring efficiency, as this is a key indicator of scaffolding quality.

We left the Hi-C scaffolded plots (now Fig. 6) exactly as they came out of a standard routine pipeline on purpose to highlight which buffers made it possible to get nicely scaffolded expected chromosomal structures in An. coluzzii. At this stage samples would have gone for curation, but we wanted to show which one performed well and would require minimal curation from the get-go. To make the interpretations a bit easier, we now added the % of reads that fall into a chromosomal scaffold for each PacBio and Hi-C combination in the upper right corner of the scaffold plots. This percentage is roughly estimated from the sizes of scaffolds that span at least one chromosome arm, and showcase how much unscaffolded "shrapnel" is left.

4. The manuscript indicates that sequencing data are not yet publicly available due to their inclusion in a larger ENA project. In line with journal policies, the authors should provide a clear plan for data release, including expected timelines and accession numbers where possible. Furthermore, additional methodological details, particularly regarding genome assembly parameters and Hi-C data processing, would improve reproducibility and transparency.

Since the manuscript's submission all raw data has been made available in their specific ENA study (PRJEB98990), and all processed data have been uploaded as part of the review process into a GigaDB study.

5. In Table 1, the inclusion of ULI scaffolding appears somewhat abrupt, as ULI sequencing is not sufficiently introduced or contextualized prior to its presentation in the table.

Although its role becomes clearer in the Results section, readers may find it difficult to fully understand its relevance at this stage. The authors may consider introducing ULI sequencing earlier in the Methods or Results, or providing a brief explanation in the table caption, to improve clarity and ensure a more coherent presentation.

We have now added a paragraph in the Methods section, DNA extraction and long read PacBio sequencing subsection, explaining in more detail on how the ULI sample was prepared and sequenced, as well as the reasoning for its inclusion.

Reviewer 2:

This manuscript presents a systematic evaluation of preservation methods for generating high-quality genomic and Hi-C data using *Anopheles coluzzii* as a model organism. The study addresses an important practical challenge in genomics, particularly for field-based sample collection where optimal preservation conditions are often difficult to achieve. The experimental design is generally well-structured, and the comparison across multiple preservation treatments provides useful insights for the community.

However, several aspects of the study require further clarification and improvement. In particular, concerns remain regarding the generalizability of the findings beyond the focal species, the robustness of the statistical analyses, and the interpretation of Hi-C results under very high sequencing coverage. Additionally, issues related to data availability and methodological transparency should be addressed to ensure reproducibility. Addressing these points would substantially strengthen the manuscript.

1. The study is conducted exclusively on *Anopheles coluzzii* under controlled laboratory conditions. While the results are promising, the applicability of these preservation strategies to other arthropods remains unclear. Given the diversity in cuticle structure, body size, and physiology across taxa, the authors should clarify the extent to which their findings can be generalized. Inclusion of additional taxa or a more explicit discussion of limitations would strengthen the manuscript.

Answered in Reviewer 1. question 1.

2. The evaluation of Hi-C data quality is based on scaffolding performance, including analyses using both high-quality and fragmented assemblies. However, the Hi-C datasets were generated at very high coverage (>100×), which may mask differences in preservation efficiency. The authors should clarify how they distinguish true preservation effects from sequencing depth-related compensation. Additional analyses using downsampled Hi-C data would provide a more realistic assessment of performance under typical conditions.

Answered in Reviewer 1. question 2.

3. I am particularly interested in the chromosome anchoring rate of the genome assemblies in the Hi-C scaffolding analysis. It would be valuable for the authors to clarify whether Hi-C data generated using different preservation methods result in differences in chromosome anchoring efficiency.

Answered in Reviewer 1. question 3.

4. The manuscript indicates that sequencing data are not yet publicly available due to their inclusion in a larger ENA project. In line with journal policies, the authors should provide a clear plan for data release, including expected timelines and accession numbers if available. Furthermore, greater detail in the Methods section—particularly regarding assembly parameters and Hi-C processing—would improve reproducibility.

Answered in Reviewer 1. question 4.

5. The column headers “Self scaffolding” and “ULI scaffolding” in Table 1 are not sufficiently clear, making it difficult for readers to fully understand the intended meaning.

We have now modified the table legend to include a more detailed description of the two column heathers, as well as a paragraph in the Methods section describing the ULI sample in better detail.

Additional reviewer reports:

Reviewer #1: This is a straightforward and valuable study. Demonstrating that breaking the cold chain is feasible has strong potential to transform how genomes can be obtained across much of biodiversity. In my view, the broader significance of this advance is somewhat underemphasized in the Introduction (Really just a short mention around lines 72-75). Based on firsthand experience in large field campaigns designed specifically to generate genomes from very small insects, I can attest that much of arthropod biodiversity (especially within small-bodied, hyperdiverse "dark taxa") stands to benefit substantially from approaches like this. The practical importance of scalable, field-friendly preservation solutions cannot be overstated.

Overall, the study is well designed, clearly presented, and addresses a real logistical bottleneck in biodiversity genomics. The conclusions are generally well supported by the data presented. I have a few suggestions that I believe would further strengthen the manuscript and increase its practical value and reproducibility.

Major / Substantive Comments

1. Framing and impact

As above, I recommend strengthening the Introduction's framing of downstream

biodiversity applications, especially for small-bodied and taxonomically challenging groups where cold-chain logistics are often the primary limiting factor. The method has particularly high relevance for large-scale efforts targeting hyperdiverse insect groups ("dark taxa"), and this applied significance could be more explicitly highlighted to broaden the paper's audience and appeal.

We thank the reviewer for their enthusiastic comments to our manuscript. We have now added a few more sentences in the Introduction and Discussion sections highlighting the importance of room temperature storage and shipment methods for arthropod and dark taxa studies.

2. Practical protocol clarity and reproducibility

Because this method is likely to be adopted by field teams, including non-specialists, it would be very helpful to include a concise protocol-style summary or workflow outlining the recommended handling steps, specimen size considerations, and timing constraints. A short practical guide or decision framework would improve reproducibility and uptake.

The detailed description (with images) on how to collect and squish specimens was published separately in a protocols.io SOP, and we have now made that more explicitly clear in the Methods section.

Relatedly, laying out any known tolerance ranges in this fashion would strengthen the paper, for example, how sensitive outcomes are to delays in processing, temperature variation, or specimen size differences under field conditions.

While we have not tested this on non-Anophelines, we have since received and sequenced several Anopheles species collected and sent from wild caught individuals, which are currently in genome curation and will be published as genome notes in the near future. We unfortunately have not done more extensive testing on storage times and sizes, but since we have adopted this storage and shipment approach on real samples already we are passively able to track this through possible sample failure or underperformance in the future.

3. Preservation benchmarking

I suggest including a summary comparison (table or figure) of yield/quality metrics across preservation treatments relative to standard cold-chain approaches. This would make performance differences easier for readers to interpret and apply.

We have now added Table 3 in the Discussion section which briefly summarizes success across our storage solutions and extraction/sequencing approach (PacBio sequencing, Hi-C sequencing, RNA extraction) and the final rating. This should make the discussion

paragraph much easier to follow and for other researchers to decide on what solution to try themselves.

4. Voucher integrity and downstream taxonomic usability

Given that many target organisms will come from taxonomically difficult groups, a short discussion of voucher integrity after treatment would be valuable. Guidance on expected morphological preservation and suitability for downstream taxonomic work would significantly increase the method's usefulness for specimen-based biodiversity genomics workflows.

As this method is extremely destructive, taxonomy should be performed initially and any valuable body part (such as mouth parts or genitalia) removed prior to tissue grinding. Because of that we were not focused on assessing morphological preservation in the specified storage solutions and subsequent freezer storage. We do highlight in the last sentence of the Discussion section that further work should be done on long term and better storage of samples for morphology, proteins and metabolites, even if they were out of scope for our manuscript.

5. Methodological Clarification

The instruction to "lightly squish" specimens to compromise the cuticle is practical, but could benefit from additional detail. Does the location of compression affect outcomes? For example, is thoracic compression preferable (to access muscle tissue), or is abdominal disruption sufficient? This may seem like a fine detail, but it matters in practice, particularly because damage to thoracic characters or terminalia can reduce taxonomic value. If there is an optimal or recommended compression location, it would be useful to specify it.

As mentioned in answer 2, we have a fully detailed protocols.io SOP on how to perform the squish approach with images, we now have a sentence highlighting this in the Methods section.

Editor Comments:

To meet the journal’s data availability requirements and ensure the reproducibility of your research findings, we kindly request that you add a Data Availability section in the revised version of your manuscript.

We have now added a Data Availability paragraph and information to each ENA raw datafile in the supplementary table. While we do not have access to the GigaDB repository to add a reference to it, we suspect these will become available once the paper is out? We have also reformatted the manuscript to better fit the GigaScience Technical Note format and added missing paragraphs at the end.

GigaScience has also published a number of relevant papers that may help contextualize your work. We have listed several recent examples below for your reference. Citing some of these articles—where appropriate in the Background or Discussion—would help frame your contribution within ongoing community efforts and strengthen the presentation of related tools and frameworks.

1. Howard C, Denton A, Jackson B W, et al. On the path to reference genomes for all biodiversity: laboratory protocols and lessons learned from processing over 2,000 species in the Sanger Tree of Life[J]. GigaScience, 2025, 14: giaf119. https://urldefense.proofpoint.com/v2/url?u=https-3A__doi.org_10.1093_gigascience_giaf119&d=DwIBaQ&c=D7ByGjS34AllFgecYw0iC6Zq7qlm8uclZFI0SqQnqBo&r=fXfLn8Z4i5uP7ApDGzQr5wdkI3tCVmWIEXk31ziY2DY&m=Sa1pZETX15nvhE759BJw9ySqjT_Qr9eO3N3q-zus-MsW0uJUK54GJQ6DdRQUoLo0&s=aCZk9jZoxIU3FwoX07GkNBOj-gl021vG_pEcnMYuu8k&e=

2. Lawniczak M K N, Kocot K M, Astrin J J, et al. Best-practice guidance for Earth BioGenome Project sample collection and processing: progress and challenges in biodiverse reference genome creation[J]. GigaScience, 2025, 14: giaf041. https://urldefense.proofpoint.com/v2/url?u=https-3A__doi.org_10.1093_gigascience_giaf041&d=DwIBaQ&c=D7ByGjS34AllFgecYw0iC6Zq7qlm8uclZFI0SqQnqBo&r=fXfLn8Z4i5uP7ApDGzQr5wdkI3tCVmWIEXk31ziY2DY&m=Sa1pZETX15nvhE759BJw9ySqjT_Qr9eO3N3q-zus-MsW0uJUK54GJQ6DdRQUoLo0&s=G00XoY13smvkFBBI0VMkI9_-c5d-0JqyQ_0miIoGVpY&e=

We have updated the two references now as at time of original submission they were only available as preprints.

Preservation solution	Treatment	Dilution factor	Volume (uL)	Diluted ng/uL	Undiluted ng/uL	Total DNA amount (ng)	Full lengtl Avg. size (bp)
100%_Ethanol_in1	1wRT_intact	5	350	0.068	0.342	119.7	32,240
100%_Ethanol_in2	1wRT_intact	5	350	0.064	0.320	112.0	31,228
100%_Ethanol_in3	1wRT_intact	5	350	0.067	0.334	116.9	40,019
100%_Ethanol_in4	1wRT_intact	7	350	0.037	0.256	89.7	46,216
100%_Ethanol_in5	1wRT_intact	6	350	0.098	0.590	206.4	41,440
100%_Ethanol_sq1	1wRT_squish	6	350	0.058	0.349	122.2	50,492
100%_Ethanol_sq2	1wRT_squish	7	350	0.091	0.636	222.5	42,728
100%_Ethanol_sq3	1wRT_squish	7	350	0.085	0.594	207.8	39,593
100%_Ethanol_sq4	1wRT_squish	5	350	0.092	0.461	161.4	34,367
100%_Ethanol_sq5	1wRT_squish	6	350	0.073	0.439	153.7	44,185
DESS_in1	1wRT_intact	1	350	0.412	0.412	144.3	17,069
DESS_in2	1wRT_intact	1	350	0.538	0.538	188.4	26,382
DESS_in3	1wRT_intact	3	350	0.263	0.789	276.2	15,729
DESS_in4	1wRT_intact	1	350	0.497	0.497	173.8	14,178
DESS_in5	1wRT_intact	1	350	0.414	0.414	144.8	17,459
DESS_sq1	1wRT_squish	3	350	0.227	0.681	238.5	51,140
DESS_sq2	1wRT_squish	1	350	0.401	0.401	140.3	59,143
DESS_sq3	1wRT_squish	3	350	0.280	0.841	294.3	40,385
DESS_sq4	1wRT_squish	3	350	0.261	0.783	274.2	45,711
DESS_sq5	1wRT_squish	1	350	0.498	0.498	174.1	30,088
EDTA_pH8_in1	1wRT_intact	7	350	0.045	0.312	109.3	35,559
EDTA_pH8_in2	1wRT_intact	7	350	0.063	0.442	154.8	17,998
EDTA_pH8_in3	1wRT_intact	5	350	0.062	0.308	107.8	27,658
EDTA_pH8_in4	1wRT_intact	6	350	0.064	0.385	134.8	29,873
EDTA_pH8_in5	1wRT_intact	4	350	0.108	0.434	151.8	27,806
EDTA_pH8_sq1	1wRT_squish	7	350	0.061	0.424	148.5	33,912
EDTA_pH8_sq2	1wRT_squish	8	350	0.083	0.666	233.0	46,320
EDTA_pH8_sq3	1wRT_squish	7	350	0.074	0.517	181.1	38,095

EDTA_pH8_sq4	1wRT_squish	6	350	0.052	0.311	109.0	46,984
EDTA_pH8_sq5	1wRT_squish	5	350	0.074	0.371	129.7	54,155
EDTA_pH9_in1	1wRT_intact	6	350	0.041	0.243	85.1	33,784
EDTA_pH9_in2	1wRT_intact	9	350	0.044	0.400	139.9	38,959
EDTA_pH9_in3	1wRT_intact	9	350	0.045	0.401	140.5	46,883
EDTA_pH9_in4	1wRT_intact	8	350	0.051	0.406	142.0	29,706
EDTA_pH9_in5	1wRT_intact	8	350	0.061	0.486	170.2	46,552
EDTA_pH9_sq1	1wRT_squish	9	350	0.029	0.265	92.6	54,129
EDTA_pH9_sq2	1wRT_squish	9	350	0.039	0.352	123.2	62,246
EDTA_pH9_sq3	1wRT_squish	10	350	0.044	0.444	155.4	53,538
EDTA_pH9_sq4	1wRT_squish	7	350	0.047	0.332	116.1	57,723
EDTA_pH9_sq5	1wRT_squish	6	350	0.036	0.214	75.0	73,594
EDTA_pH10_in1	1wRT_intact	6	350	0.055	0.330	115.5	31,634
EDTA_pH10_in2	1wRT_intact	6	350	0.045	0.269	94.1	40,523
EDTA_pH10_in3	1wRT_intact	6	350	0.035	0.212	74.3	44,195
EDTA_pH10_in4	1wRT_intact	5	350	0.036	0.179	62.5	52,308
EDTA_pH10_in5	1wRT_intact	4	350	0.044	0.174	60.9	40,226
EDTA_pH10_sq1	1wRT_squish	6	350	0.068	0.407	142.6	33,790
EDTA_pH10_sq2	1wRT_squish	6	350	0.049	0.296	103.7	66,678
EDTA_pH10_sq3	1wRT_squish	6	350	0.050	0.300	105.0	41,408
EDTA_pH10_sq4	1wRT_squish	7	350	0.061	0.424	148.5	55,649
EDTA_pH10_sq5	1wRT_squish	5	350	0.043	0.215	75.1	59,675
Allprotect_in1	1wRT_intact	1	350	0.401	0.401	140.2	29,192
Allprotect_in2	1wRT_intact	1	350	0.495	0.495	173.4	20,230
Allprotect_in3	1wRT_intact	3	350	0.246	0.738	258.3	28,668
Allprotect_in4	1wRT_intact	1	350	0.401	0.401	140.4	27,500
Allprotect_in5	1wRT_intact	4	350	0.231	0.924	323.5	26,027
Allprotect_sq1	1wRT_squish	3	350	0.330	0.989	346.1	34,538
Allprotect_sq2	1wRT_squish	4	350	0.261	1.044	365.4	43,934
Allprotect_sq3	1wRT_squish	3	350	0.246	0.737	257.9	31,622
Allprotect_sq4	1wRT_squish	1	350	0.574	0.574	201.0	32,036
Allprotect_sq5	1wRT_squish	1	350	0.562	0.562	196.7	30,686

100%_Ethanol_in1	ff-1wRT_intact	6	350	0.078	0.467	163.6	32,320
100%_Ethanol_in2	ff-1wRT_intact	7	350	0.073	0.512	179.3	26,738
100%_Ethanol_in3	ff-1wRT_intact	5	350	0.078	0.390	136.5	30,040
100%_Ethanol_in4	ff-1wRT_intact	6	350	0.055	0.332	116.3	35,933
100%_Ethanol_in5	ff-1wRT_intact	1	350	0.220	0.220	76.9	60,383
100%_Ethanol_sq1	ff-1wRT_squish	6	350	0.088	0.529	185.2	23,061
100%_Ethanol_sq2	ff-1wRT_squish	4	350	0.080	0.319	111.6	33,639
100%_Ethanol_sq3	ff-1wRT_squish	7	350	0.078	0.546	191.1	21,280
100%_Ethanol_sq4	ff-1wRT_squish	8	350	0.124	0.993	347.5	23,790
100%_Ethanol_sq5	ff-1wRT_squish	8	350	0.085	0.678	237.4	29,698
DESS_in1	ff-1wRT_intact	1	350	0.413	0.413	144.7	9,906
DESS_in2	ff-1wRT_intact	1	350	0.550	0.550	192.6	11,993
DESS_in3	ff-1wRT_intact	3	350	0.255	0.764	267.4	15,684
DESS_in4	ff-1wRT_intact	1	350	0.390	0.390	136.6	15,672
DESS_in5	ff-1wRT_intact	1	350	0.742	0.742	259.6	27,657
DESS_sq1	ff-1wRT_squish	3	350	0.290	0.870	304.5	43,123
DESS_sq2	ff-1wRT_squish	5	350	0.218	1.092	382.2	39,099
DESS_sq3	ff-1wRT_squish	4	350	0.309	1.237	432.9	30,214
DESS_sq4	ff-1wRT_squish	3	350	0.224	0.671	235.0	64,558
DESS_sq5	ff-1wRT_squish	4	350	0.249	0.995	348.2	25,763
EDTA_pH8_in1	ff-1wRT_intact	5	350	0.042	0.209	73.2	20,734
EDTA_pH8_in2	ff-1wRT_intact	7	350	0.066	0.463	162.2	37,393
EDTA_pH8_in3	ff-1wRT_intact	5	350	0.061	0.303	106.1	24,623
EDTA_pH8_in4	ff-1wRT_intact	8	350	0.041	0.325	113.7	23,737
EDTA_pH8_in5	ff-1wRT_intact	5	350	0.066	0.331	115.9	31,167
EDTA_pH8_sq1	ff-1wRT_squish	10	350	0.064	0.642	224.7	58,075
EDTA_pH8_sq2	ff-1wRT_squish	9	350	0.049	0.440	154.0	45,492
EDTA_pH8_sq3	ff-1wRT_squish	8	350	0.052	0.414	145.0	55,738
EDTA_pH8_sq4	ff-1wRT_squish	7	350	0.062	0.432	151.2	36,661
EDTA_pH8_sq5	ff-1wRT_squish	10	350	0.069	0.692	242.2	66,598
EDTA_pH9_in1	ff-1wRT_intact	7	350	0.042	0.296	103.6	39,953
EDTA_pH9_in2	ff-1wRT_intact	7	350	0.050	0.351	122.7	30,282

EDTA_pH9_in3	ff-1wRT_intact	7	350	0.057	0.398	139.4	36,763
EDTA_pH9_in4	ff-1wRT_intact	9	350	0.046	0.410	143.6	39,051
EDTA_pH9_in5	ff-1wRT_intact	9	350	0.052	0.464	162.5	35,857
EDTA_pH9_sq1	ff-1wRT_squish	9	350	0.034	0.304	106.5	63,562
EDTA_pH9_sq2	ff-1wRT_squish	6	350	0.048	0.290	101.6	58,259
EDTA_pH9_sq3	ff-1wRT_squish	9	350	0.046	0.418	146.2	40,888
EDTA_pH9_sq4	ff-1wRT_squish	11	350	0.069	0.762	266.8	68,297
EDTA_pH9_sq5	ff-1wRT_squish	8	350	0.042	0.338	118.4	61,586
EDTA_pH10_in1	ff-1wRT_intact	10	350	0.036	0.364	127.4	70,073
EDTA_pH10_in2	ff-1wRT_intact	9	350	0.026	0.233	81.6	34,231
EDTA_pH10_in3	ff-1wRT_intact	9	350	0.035	0.311	109.0	48,739
EDTA_pH10_in4	ff-1wRT_intact	7	350	0.060	0.422	147.7	42,657
EDTA_pH10_in5	ff-1wRT_intact	7	350	0.047	0.328	114.9	48,775
EDTA_pH10_sq1	ff-1wRT_squish	8	350	0.046	0.365	127.7	44,248
EDTA_pH10_sq2	ff-1wRT_squish	11	350	0.041	0.455	159.4	70,433
EDTA_pH10_sq3	ff-1wRT_squish	6	350	0.043	0.258	90.3	41,785
EDTA_pH10_sq4	ff-1wRT_squish	8	350	0.046	0.371	129.9	68,935
EDTA_pH10_sq5	ff-1wRT_squish	12	350	0.031	0.366	128.1	93,743
Allprotect_in1	ff-1wRT_intact	3	350	0.277	0.832	291.3	26,577
Allprotect_in2	ff-1wRT_intact	3	350	0.267	0.800	280.1	31,273
Allprotect_in3	ff-1wRT_intact	3	350	0.199	0.597	209.0	35,070
Allprotect_in4	ff-1wRT_intact	1	350	0.485	0.485	169.7	26,172
Allprotect_in5	ff-1wRT_intact	1	350	0.466	0.466	163.0	25,932
Allprotect_sq1	ff-1wRT_squish	3	350	0.237	0.710	248.4	42,174
Allprotect_sq2	ff-1wRT_squish	4	350	0.183	0.730	255.5	68,131
Allprotect_sq3	ff-1wRT_squish	4	350	0.221	0.884	309.5	42,977
Allprotect_sq4	ff-1wRT_squish	3	350	0.222	0.667	233.3	43,941
Allprotect_sq5	ff-1wRT_squish	3	350	0.220	0.659	230.7	41,031
snapfrozen_control1	control	1	350	0.414	0.414	144.8	22,779
snapfrozen_control2	control	1	350	0.543	0.543	189.9	28,947
snapfrozen_control3	control	1	350	0.573	0.573	200.6	27,451
snapfrozen_control4	control	3	350	0.222	0.666	233.1	36,895

snapfrozen_control5	control	1	350	0.136	0.136	47.6	55,673
**squish to squish comparison**							
100%_Ethanol_sq1	1wRT_squish	6	350	0.058	0.349	122.2	50,492
100%_Ethanol_sq2	1wRT_squish	7	350	0.091	0.636	222.5	42,728
100%_Ethanol_sq3	1wRT_squish	7	350	0.085	0.594	207.8	39,593
100%_Ethanol_sq4	1wRT_squish	5	350	0.092	0.461	161.4	34,367
100%_Ethanol_sq5	1wRT_squish	6	350	0.073	0.439	153.7	44,185
100%_Ethanol_sq1	ff-1wRT_squish	6	350	0.088	0.529	185.2	23,061
100%_Ethanol_sq2	ff-1wRT_squish	4	350	0.080	0.319	111.6	33,639
100%_Ethanol_sq3	ff-1wRT_squish	7	350	0.078	0.546	191.1	21,280
100%_Ethanol_sq4	ff-1wRT_squish	8	350	0.124	0.993	347.5	23,790
100%_Ethanol_sq5	ff-1wRT_squish	8	350	0.085	0.678	237.4	29,698
DESS_sq1	1wRT_squish	3	350	0.227	0.681	238.5	51,140
DESS_sq2	1wRT_squish	1	350	0.401	0.401	140.3	59,143
DESS_sq3	1wRT_squish	3	350	0.280	0.841	294.3	40,385
DESS_sq4	1wRT_squish	3	350	0.261	0.783	274.2	45,711
DESS_sq5	1wRT_squish	1	350	0.498	0.498	174.1	30,088
DESS_sq1	ff-1wRT_squish	3	350	0.290	0.870	304.5	43,123
DESS_sq2	ff-1wRT_squish	5	350	0.218	1.092	382.2	39,099
DESS_sq3	ff-1wRT_squish	4	350	0.309	1.237	432.9	30,214
DESS_sq4	ff-1wRT_squish	3	350	0.224	0.671	235.0	64,558
DESS_sq5	ff-1wRT_squish	4	350	0.249	0.995	348.2	25,763
EDTA_pH8_sq1	1wRT_squish	7	350	0.061	0.424	148.5	33,912
EDTA_pH8_sq2	1wRT_squish	8	350	0.083	0.666	233.0	46,320
EDTA_pH8_sq3	1wRT_squish	7	350	0.074	0.517	181.1	38,095
EDTA_pH8_sq4	1wRT_squish	6	350	0.052	0.311	109.0	46,984
EDTA_pH8_sq5	1wRT_squish	5	350	0.074	0.371	129.7	54,155
EDTA_pH8_sq1	ff-1wRT_squish	10	350	0.064	0.642	224.7	58,075
EDTA_pH8_sq2	ff-1wRT_squish	9	350	0.049	0.440	154.0	45,492
EDTA_pH8_sq3	ff-1wRT_squish	8	350	0.052	0.414	145.0	55,738
EDTA_pH8_sq4	ff-1wRT_squish	7	350	0.062	0.432	151.2	36,661

EDTA_pH8_sq5	ff-1wRT_squish	10	350	0.069	0.692	242.2	66,598
EDTA_pH9_sq1	1wRT_squish	9	350	0.029	0.265	92.6	54,129
EDTA_pH9_sq2	1wRT_squish	9	350	0.039	0.352	123.2	62,246
EDTA_pH9_sq3	1wRT_squish	10	350	0.044	0.444	155.4	53,538
EDTA_pH9_sq4	1wRT_squish	7	350	0.047	0.332	116.1	57,723
EDTA_pH9_sq5	1wRT_squish	6	350	0.036	0.214	75.0	73,594
EDTA_pH9_sq1	ff-1wRT_squish	9	350	0.034	0.304	106.5	63,562
EDTA_pH9_sq2	ff-1wRT_squish	6	350	0.048	0.290	101.6	58,259
EDTA_pH9_sq3	ff-1wRT_squish	9	350	0.046	0.418	146.2	40,888
EDTA_pH9_sq4	ff-1wRT_squish	11	350	0.069	0.762	266.8	68,297
EDTA_pH9_sq5	ff-1wRT_squish	8	350	0.042	0.338	118.4	61,586
EDTA_pH10_sq1	1wRT_squish	6	350	0.068	0.407	142.6	33,790
EDTA_pH10_sq2	1wRT_squish	6	350	0.049	0.296	103.7	66,678
EDTA_pH10_sq3	1wRT_squish	6	350	0.050	0.300	105.0	41,408
EDTA_pH10_sq4	1wRT_squish	7	350	0.061	0.424	148.5	55,649
EDTA_pH10_sq5	1wRT_squish	5	350	0.043	0.215	75.1	59,675
EDTA_pH10_sq1	ff-1wRT_squish	8	350	0.046	0.365	127.7	44,248
EDTA_pH10_sq2	ff-1wRT_squish	11	350	0.041	0.455	159.4	70,433
EDTA_pH10_sq3	ff-1wRT_squish	6	350	0.043	0.258	90.3	41,785
EDTA_pH10_sq4	ff-1wRT_squish	8	350	0.046	0.371	129.9	68,935
EDTA_pH10_sq5	ff-1wRT_squish	12	350	0.031	0.366	128.1	93,743
Allprotect_sq1	1wRT_squish	3	350	0.330	0.989	346.1	34,538
Allprotect_sq2	1wRT_squish	4	350	0.261	1.044	365.4	43,934
Allprotect_sq3	1wRT_squish	3	350	0.246	0.737	257.9	31,622
Allprotect_sq4	1wRT_squish	1	350	0.574	0.574	201.0	32,036
Allprotect_sq5	1wRT_squish	1	350	0.562	0.562	196.7	30,686
Allprotect_sq1	ff-1wRT_squish	3	350	0.237	0.710	248.4	42,174
Allprotect_sq2	ff-1wRT_squish	4	350	0.183	0.730	255.5	68,131
Allprotect_sq3	ff-1wRT_squish	4	350	0.221	0.884	309.5	42,977
Allprotect_sq4	ff-1wRT_squish	3	350	0.222	0.667	233.3	43,941
Allprotect_sq5	ff-1wRT_squish	3	350	0.220	0.659	230.7	41,031

h range				DNA above 5 kbp in length				
Avg. DNA amount (ng)	Degree of freedom	t-test	p-value	Diluted ng/uL	Undiluted ng/uL	Total DNA amount (ng)	Avg. size (bp)	Avg. DNA amount (ng)
				0.055	0.274	95.9	39,847	
				0.043	0.214	74.7	46,451	
128.94				0.057	0.287	100.3	46,415	102.88
				0.029	0.204	71.3	57,716	
	7.9364	-1.6379	0.1404	0.082	0.492	172.2	49,353	
				0.052	0.311	108.8	56,444	
				0.078	0.544	190.4	49,609	
173.50				0.073	0.512	179.1	45,593	147.75
				0.073	0.365	127.6	43,070	
				0.063	0.380	132.9	50,730	
				0.145	0.145	50.9	47,645	
				0.239	0.239	83.8	58,809	
185.52				0.085	0.255	89.3	47,500	71.91
				0.206	0.206	71.9	33,078	
	7.7248	-1.0200	0.3386	0.182	0.182	63.7	38,831	
				0.167	0.501	175.2	69,372	
				0.339	0.339	118.8	69,660	
224.27				0.159	0.477	167.1	70,532	145.13
				0.166	0.497	173.8	71,897	
				0.260	0.260	90.8	57,117	
				0.034	0.236	82.6	46,459	
				0.032	0.222	77.7	34,551	
131.70				0.043	0.213	74.4	39,464	89.48
				0.048	0.286	100.0	39,574	
	5.6414	-1.1928	0.2807	0.081	0.322	112.8	36,874	
				0.040	0.281	98.5	50,181	
				0.069	0.554	193.8	55,256	
160.23				0.052	0.363	126.9	53,567	124.52

				0.044	0.265	92.8	54,909	
				0.063	0.316	110.6	63,159	
				0.027	0.164	57.5	49,098	
				0.036	0.320	112.1	48,239	
135.52				0.034	0.310	108.4	60,334	104.61
				0.038	0.302	105.6	39,377	
	7.9994	1.1825	0.2710	0.050	0.398	139.4	56,411	
				0.025	0.222	77.8	64,065	
				0.034	0.305	106.8	71,378	
112.46				0.034	0.342	119.7	68,870	93.72
				0.039	0.274	96.0	69,447	
				0.033	0.195	68.3	80,424	
				0.035	0.209	73.1	49,168	
				0.029	0.176	61.7	61,109	
81.46				0.025	0.151	52.9	61,424	54.72
				0.028	0.139	48.5	66,847	
	7.4756	-1.9590	0.0883	0.027	0.107	37.4	64,648	
				0.045	0.272	95.1	49,832	
				0.044	0.262	91.8	75,008	
114.98				0.035	0.209	73.1	58,976	90.16
				0.054	0.375	131.3	62,661	
				0.034	0.170	59.5	74,956	
				0.298	0.298	104.3	38,916	
				0.257	0.257	89.9	38,424	
207.16				0.151	0.453	158.7	46,371	135.96
				0.303	0.303	106.1	35,989	
	7.9960	-1.3079	0.2273	0.158	0.631	220.9	37,544	
				0.216	0.649	227.0	52,531	
				0.213	0.851	297.8	53,616	
273.43				0.175	0.526	184.1	43,996	197.47
				0.432	0.432	151.1	42,377	
				0.364	0.364	127.4	46,969	

				0.068	0.409	143.0	36,676	
				0.056	0.391	136.7	34,416	
134.54				0.070	0.350	122.5	33,291	115.26
				0.049	0.295	103.3	40,177	
	5.6464	-1.8678	0.1141	0.202	0.202	70.7	65,486	
				0.065	0.391	136.9	30,474	
				0.070	0.278	97.4	38,180	
214.56				0.059	0.415	145.3	27,358	173.20
				0.103	0.823	288.1	28,194	
				0.071	0.566	198.2	35,156	
				0.126	0.126	44.1	31,433	
				0.198	0.198	69.2	32,318	
200.19				0.100	0.300	105.1	38,935	86.21
				0.164	0.164	57.5	36,631	
	7.6984	-3.2200	*0.0129*	0.443	0.443	155.2	45,978	
				0.228	0.683	238.9	54,747	
				0.154	0.771	269.9	54,814	
340.55				0.171	0.684	239.4	53,852	212.32
				0.164	0.491	171.7	88,096	
				0.101	0.405	141.8	62,542	
				0.030	0.148	51.6	28,610	
				0.049	0.342	119.6	50,121	
114.18				0.046	0.232	81.0	31,603	87.27
				0.031	0.246	86.0	30,699	
	7.1019	-2.7595	*0.0277*	0.056	0.281	98.2	36,390	
				0.056	0.562	196.7	65,991	
				0.041	0.365	127.9	54,236	
183.43				0.045	0.362	126.6	63,519	157.00
				0.047	0.327	114.4	47,806	
				0.063	0.627	219.5	73,152	
				0.035	0.244	85.5	47,994	
				0.035	0.246	86.2	42,401	

134.39				0.047	0.331	115.9	43,816	107.93
				0.038	0.342	119.7	46,420	
				0.042	0.378	132.3	43,604	
	4.8324	-0.4184	0.6936	0.029	0.258	90.4	74,490	
				0.043	0.258	90.3	65,245	
147.90				0.036	0.327	114.3	51,618	128.71
				0.063	0.694	242.9	74,743	
				0.038	0.302	105.6	68,835	
				0.031	0.312	109.2	81,307	
				0.015	0.136	47.6	57,710	
116.12				0.024	0.216	75.6	69,591	88.96
				0.048	0.337	118.1	52,917	
				0.039	0.270	94.3	59,075	
	7.9996	-0.7088	0.4986	0.037	0.295	103.3	54,022	
				0.035	0.382	133.6	83,484	
127.08				0.036	0.215	75.2	49,874	106.77
				0.041	0.324	113.4	78,654	
				0.026	0.310	108.4	110,549	
				0.168	0.503	176.0	43,506	
				0.135	0.406	142.1	61,322	
222.60				0.151	0.452	158.3	46,072	141.30
				0.279	0.279	97.8	44,673	
				0.378	0.378	132.3	31,545	
	6.0766	-1.0772	0.3223	0.181	0.543	190.2	54,867	
				0.165	0.661	231.3	75,136	
255.49				0.193	0.771	269.9	49,065	205.74
				0.169	0.507	177.6	57,414	
				0.152	0.457	159.8	59,027	
				0.247	0.247	86.6	37,512	
				0.343	0.343	120.1	45,403	
163.21				0.313	0.313	109.7	49,686	110.59
				0.185	0.555	194.4	43,998	

				0.121	0.121	42.3	62,418	
[redacted]								
				0.052	0.311	108.8	56,444	
				0.078	0.544	190.4	49,609	
173.50				0.073	0.512	179.1	45,593	147.75
				0.073	0.365	127.6	43,070	
	5.7002	0.9554	0.3781	0.063	0.380	132.9	50,730	
				0.065	0.391	136.9	30,474	
				0.070	0.278	97.4	38,180	
214.56				0.059	0.415	145.3	27,358	173.20
				0.103	0.823	288.1	28,194	
				0.071	0.566	198.2	35,156	
				0.167	0.501	175.2	69,372	
				0.339	0.339	118.8	69,660	
224.27				0.159	0.477	167.1	70,532	145.13
				0.166	0.497	173.8	71,897	
	7.8455	2.6027	*0.0320*	0.260	0.260	90.8	57,117	
				0.228	0.683	238.9	54,747	
				0.154	0.771	269.9	54,814	
340.55				0.171	0.684	239.4	53,852	212.32
				0.164	0.491	171.7	88,096	
				0.101	0.405	141.8	62,542	
				0.040	0.281	98.5	50,181	
				0.069	0.554	193.8	55,256	
160.23				0.052	0.363	126.9	53,567	124.52
				0.044	0.265	92.8	54,909	
	7.9806	0.7742	0.4611	0.063	0.316	110.6	63,159	
				0.056	0.562	196.7	65,991	
				0.041	0.365	127.9	54,236	
183.43				0.045	0.362	126.6	63,519	157.00
				0.047	0.327	114.4	47,806	

				0.063	0.627	219.5	73,152	
				0.025	0.222	77.8	64,065	
				0.034	0.305	106.8	71,378	
112.46				0.034	0.342	119.7	68,870	93.72
				0.039	0.274	96.0	69,447	
				0.033	0.195	68.3	80,424	
	5.5378	1.0536	0.3358	0.029	0.258	90.4	74,490	
				0.043	0.258	90.3	65,245	
147.90				0.036	0.327	114.3	51,618	128.71
				0.063	0.694	242.9	74,743	
				0.038	0.302	105.6	68,835	
				0.045	0.272	95.1	49,832	
				0.044	0.262	91.8	75,008	
114.98				0.035	0.209	73.1	58,976	90.16
				0.054	0.375	131.3	62,661	
				0.034	0.170	59.5	74,956	
	7.6554	0.6925	0.5091	0.037	0.295	103.3	54,022	
				0.035	0.382	133.6	83,484	
127.08				0.036	0.215	75.2	49,874	106.77
				0.041	0.324	113.4	78,654	
				0.026	0.310	108.4	110,549	
				0.216	0.649	227.0	52,531	
				0.213	0.851	297.8	53,616	
273.43				0.175	0.526	184.1	43,996	197.47
				0.432	0.432	151.1	42,377	
				0.364	0.364	127.4	46,969	
	5.2664	-0.4695	0.6575	0.181	0.543	190.2	54,867	
				0.165	0.661	231.3	75,136	
255.49				0.193	0.771	269.9	49,065	205.74
				0.169	0.507	177.6	57,414	
				0.152	0.457	159.8	59,027	

			Average fragment size (full range)			
Degree of freedom	t-test	p-value	Avg. DNA size (bp)	Degree of freedom	t-test	p-value
7.8302	-1.8635	0.1002	38,229<br>42,273	7.9615	-1.0386	0.3295
5.2647	-3.9702	*0.0096*	18,163<br>45,293	5.4606	-5.0707	*0.0030*
5.2513	-1.7801	0.1324	27,779<br>43,893	7.6136	-3.5393	*0.0083*

7.2031	0.6721	0.5225	39,177<br>60,246	7.9539	-4.1989	*0.0030*
5.8620	-2.6119	*0.0409*	41,777<br>51,440	6.2406	-1.3995	0.2094
7.6481	-1.5901	0.1522	26,323<br>34,563	6.9666	-2.8245	*0.0257*

5.2277	-1.6365	0.1601	37,083<br>26,294	5.1619	1.6739	0.1532
7.7647	-4.0524	*0.0039*	16,182<br>40,551	5.5928	-3.2863	*0.0185*
6.0315	-2.9035	*0.0271*	27,531<br>52,513	6.3939	-4.1641	*0.0051*

			36,381			
4.8365	-0.6833	0.5258		5.0249	-4.4324	*0.0022*
			58,518			
			48,895			
7.4061	-1.1318	0.2930		6.6724	-1.3261	0.2284
			63,829			
			29,005			
6.9394	-2.7009	*0.0309*		4.9728	-3.4210	*0.0190*
			47,651			
			34,349			

[redacted]						
5.7349	0.6977	0.5126	42,273 / 26,294	7.8555	-4.5333	*0.0020*
7.2554	2.2897	0.0546	45,293 / 40,551	7.3057	-0.5684	0.5868
7.8201	1.1580	0.2810	43,893 / 52,513	7.0801	1.3678	0.2132

4.8291	1.1510	0.3035	$\frac{60,246}{58,518}$	7.5669	-0.2895	0.7800
7.5331	1.0802	0.3134	$\frac{51,440}{63,829}$	6.7486	1.0942	0.3114
6.9295	0.2291	0.8254	$\frac{34,563}{47,651}$	5.6988	2.3015	0.0633

Preservation solution	Treatment	Dilution factor	Volume (µl)	Diluted ng/uL
100%_Ethanol_sq1	1wRT_squish	4	350	0.607
100%_Ethanol_sq2	1wRT_squish	5	350	0.313
100%_Ethanol_sq3	1wRT_squish	2	350	0.384
100%_Ethanol_sq4	1wRT_squish	5	350	0.416
100%_Ethanol_sq5	1wRT_squish	5	350	0.417
DESS_sq1	1wRT_squish	3	350	0.731
DESS_sq2	1wRT_squish	3	350	0.574
DESS_sq3	1wRT_squish	3	350	0.484
DESS_sq4	1wRT_squish	4	350	0.506
DESS_sq5	1wRT_squish	5	350	0.550
EDTA_pH8_sq1	1wRT_squish	4	350	0.827
EDTA_pH8_sq2	1wRT_squish	4	350	0.427
EDTA_pH8_sq3	1wRT_squish	4	350	0.581
EDTA_pH8_sq4	1wRT_squish	4	350	0.546
EDTA_pH8_sq5	1wRT_squish	3	350	0.590
Allprotect_sq1	1wRT_squish	4	350	0.628
Allprotect_sq2	1wRT_squish	3	350	0.545
Allprotect_sq3	1wRT_squish	3	350	0.507
Allprotect_sq4	1wRT_squish	4	350	0.440
Allprotect_sq5	1wRT_squish	4	350	0.360
RNAlater_sq1	1wRT_squish	4	350	0.521
RNAlater_sq2	1wRT_squish	5	350	0.515
RNAlater_sq3	1wRT_squish	3	350	0.541
RNAlater_sq4	1wRT_squish	5	350	0.445
RNAlater_sq5	1wRT_squish	4	350	0.513
snapfrozen_control1	control	4	350	0.492
snapfrozen_control2	control	3	350	0.565
snapfrozen_control3	control	3	350	0.548
snapfrozen_control4	control	2	350	0.653
snapfrozen_control5	control	4	350	0.482

Preservation solution	Treatment	Qubit ng/µl	Volume (µl)	Total RNA (ng)
100%_Ethanol_sq6	1wRT_squish	3.1	45	137.7
100%_Ethanol_sq7	1wRT_squish	2.8	45	124.2
100%_Ethanol_sq8	1wRT_squish	3.1	45	139.1

100%_Ethanol_sq9	1wRT_squish	3.4	45	151.2
DESS_sq6	1wRT_squish	9.3	45	419.9
DESS_sq7	1wRT_squish	11.5	45	517.5
DESS_sq8	1wRT_squish	8.6	45	387.0
DESS_sq9	1wRT_squish	6.1	45	272.3
EDTA_pH8_sq6	1wRT_squish	7.9	45	354.2
EDTA_pH8_sq7	1wRT_squish	5.0	45	225.5
EDTA_pH8_sq8	1wRT_squish	2.3	45	103.1
EDTA_pH8_sq9	1wRT_squish	6.7	45	302.9
Allprotect_sq6	1wRT_squish	3.6	45	163.4
Allprotect_sq7	1wRT_squish	3.2	45	144.0
Allprotect_sq8	1wRT_squish	4.5	45	204.3
Allprotect_sq9	1wRT_squish	3.0	45	136.4
RNAlater_sq6	1wRT_squish	5.7	45	257.9
RNAlater_sq7	1wRT_squish	n/a	n/a	n/a
RNAlater_sq8	1wRT_squish	6.3	45	282.6
RNAlater_sq9	1wRT_squish	5.7	45	257.0
snapfrozen_control6	control	7.3	45	329.9
snapfrozen_control7	control	6.1	45	274.1
snapfrozen_control8	control	6.6	45	297.5
snapfrozen_control9	control	5.1	45	230.9

Full length range					
Undiluted ng/uL	Total DNA amount (ng)	Avg. size (bp)	Avg. DNA amount (ng)	Degree of freedom	t-test
2.426	849.1	31,949			
1.564	547.2	43,789			
0.768	268.7	53,551	624.5	5.3141	-0.2438
2.080	727.8	51,576			
2.085	729.6	39,254			
2.193	767.7	34,653			
1.721	602.2	44,819			
1.452	508.2	37,136	709.9	6.1387	-1.2771
2.026	709.0	38,390			
2.750	962.3	15,674			
3.308	1157.8	27,812			
1.707	597.4	38,526			
2.322	812.7	39,955	790.2	5.3263	-1.7664
2.183	764.0	59,724			
1.769	619.3	30,405			
2.511	878.8	22,126			
1.635	572.1	33,840			
1.521	532.5	33,500	620.7	6.6721	-0.2899
1.760	615.9	33,259			
1.441	504.4	35,565			
2.085	729.8	33,006			
2.576	901.6	21,918			
1.622	567.7	33,826	739.0	7.5241	-2.0773
2.223	778.1	41,029			
2.051	717.9	23,938			
1.968	688.9	23,233			
1.696	593.5	26,487			
1.643	575.1	35,232	597.8		
1.306	457.0	28,919			
1.928	674.7	23,589			

Total RNA					
Tapestation avg. size (nt)	Avg. RNA amount (ng)	Degree of freedom	t-test	p value	Qubit (ng/μl)
3,138					3.7
3,164	138.0	3.4198	6.7312	*0.0044*	2.6
3,164					3.4

6,566					3.6
4,507	399.2	3.9890	-2.1229	0.1012	7.5
2,919					6.9
2,485					7.9
3,827					5.5
3,344	246.4	3.8544	0.6275	0.5656	11.5
4,793					8.1
3,356					5.6
3,390					8.9
3,588	162.0	5.4903	4.6952	*0.0042*	6.1
3,857					2.9
3,925					3.9
3,731					2.4
3,836	265.8	3.9016	0.7682	0.4862	5.8
n/a					n/a
3,498					6.5
3,295					5.8
4,153	283.1				8.9
3,897					6.3
3,843					5.7
3,721					5.6

p value	Diluted ng/uL	Undiluted ng/uL	Total DNA amount (ng)	Avg. size (bp)	Avg. DNA amount (ng)
	DNA above 5 kbp in length				
0.8165	0.470	1.882	658.6	40,994	452.9
	0.230	1.149	402.2	59,520	
	0.265	0.530	185.5	77,308	
	0.290	1.452	508.0	73,588	
	0.292	1.459	510.5	55,771	
0.2477	0.554	1.663	582.1	45,361	485.3
	0.426	1.277	447.0	60,107	
	0.346	1.039	363.5	51,504	
	0.315	1.259	440.7	61,236	
	0.339	1.695	593.3	24,787	
0.1340	0.604	2.417	845.9	37,261	620.7
	0.320	1.280	447.9	50,747	
	0.509	2.035	712.2	45,317	
	0.486	1.945	680.8	66,836	
	0.397	1.191	416.9	44,461	
0.7807	0.511	2.046	716.0	26,777	488.9
	0.435	1.304	456.3	42,087	
	0.351	1.054	368.8	47,578	
	0.339	1.357	474.9	42,723	
	0.306	1.224	428.5	41,596	
0.0736	0.389	1.556	544.6	43,837	520.1
	0.338	1.690	591.5	32,675	
	0.386	1.158	405.4	46,976	
	0.325	1.625	568.6	55,814	
	0.350	1.402	490.6	34,542	
	0.403	1.612	564.1	27,970	471.8
	0.458	1.373	480.7	32,280	
	0.424	1.271	444.7	45,202	
	0.520	1.040	364.1	35,845	
	0.361	1.444	505.4	30,979	

Volume (µl)	Total RNA (ng)	Avg. RNA amount (ng)	Degree of freedom	t-test	p value
		RNA after DNase treatment			
30	110.7	99.4	3.5758	4.0959	*0.0187*
30	78.6				
30	100.5				

30	107.7				
30	225.9	208.5	5.3501	-0.3253	0.7573
30	206.1				
30	237.9				
30	164.1				
30	345.0	255.4	5.0695	-1.2878	0.2535
30	242.1				
30	166.5				
30	267.9				
30	182.7	115.1	5.9893	2.5025	*0.0464*
30	87.6				
30	116.7				
30	73.2				
30	173.4	180.4	3.4591	0.5323	0.6269
n/a	n/a				
30	194.1				
30	173.7				
30	267.9	199.3			
30	188.7				
30	171.3				
30	169.2				

Degree of freedom	t-test	p value	Average fragment size (full range)			
			Avg. DNA size (bp)	Degree of freedom	t-test	p value
7.8496	0.1555	0.8804	44,024	6.2289	-3.6391	*0.0102*
6.7572	-0.2675	0.7970	34,134	5.5364	-1.2349	0.2667
7.6231	-1.5450	0.1628	39,284	5.1966	-1.9578	0.1054
7.8739	-0.2775	0.7885	31,658	7.9266	-1.2761	0.2381
5.4997	-0.8615	0.4249	30,743	6.7274	-0.7875	0.4578
			27,492			

Average fragment size (full range)			
Avg. RNA length (nt)	Degree of freedom	t-test	p value
4,008	3.0683	-0.1219	0.9106

3,435	3.2406	1.0133	0.3805
3,721	3.3866	0.4953	0.6507
3,775	5.7685	1.0924	0.3182
3,543	3.3064	1.9795	0.1336
3,904			

PacBio sequencing run		Hi-C sequencing run*	
**ToL ID**	**ENA ID**	**ToL ID**	**ENA ID**
idAnoColu456	ERR15760899		
not sequenced			
not sequenced		idAnoColu455	ERR15865280
not sequenced			
not sequenced			
not sequenced			
not sequenced			
not sequenced		idAnoColu425	ERR15865277
idAnoColu429	ERR15760895		
not sequenced			
not sequenced			
not sequenced			
not sequenced		idAnoColu435	ERR15865278
idAnoColu439	ERR15760896		
not sequenced			
idAnoColu416	ERR15760897		
not sequenced			
not sequenced		idAnoColu415	ERR15865279
not sequenced			
not sequenced			
not sequenced			
not sequenced			
not sequenced		idAnoColu465	ERR15865281
not sequenced			
idAnoColu470	ERR15760898		
not sequenced			
not sequenced			
idAnoColu448	ERR15760894	idAnoColu445	ERR15865276
not sequenced			
not sequenced			

* separate individual to the one used for PacBio sequencing from the same laboratory strain
