# Supplementary material for: Breaking the cold chain: solutions for room temperature preservation of mosquitoes leading to high quality reference genomes
Source: Gigascience. 2026 May 22;15:giag061. doi: 10.1093/gigascience/giag061 (PMC13221836; doi:10.1093/gigascience/giag061)
Supplement: giag061_Authors_Response_To_Reviewer_Comments_original_submission [file giag061_authors_response_to_reviewer_comments_original_submission.pdf]

Author's Response To Reviewer Comments

- We thank the reviewers and editor for their insightful comments, and have included most of their suggestions into the manu
why that is out of the scope of this paper. All new sentences and paragraphs are highlighted with red text in the resubmission
in a GigaScience Technical Note publication. Below we go through each comment separately.

Reviewer 1:
This study uses Anopheles coluzzii as a model to systematically evaluate preservation methods for generating high-quality ge
approaches that eliminate the need for cold-chain transport is a particularly important step forward for field-based genomics,
provides useful experimental evidence and practical guidance with broad potential value. That said, several aspects of the ma
1. The study is conducted exclusively on Anopheles coluzzii under controlled laboratory conditions. While the results are prom
remains unclear. Given the substantial diversity in cuticle structure, body size, and physiological properties across taxa, the a
of additional taxa would be ideal; alternatively, a more thorough discussion of potential constraints would improve the manus
- We have now added a small paragraph explaining the limitations of the result interpretation when applying these methods t
colony, it was the easiest and most minimally variable organism we could use across all tests to minimize internal sample bia
Anopheles samples that were sent squished at room temperature, they are still going through curation but will be published s

2. The evaluation of Hi-C data quality is primarily based on scaffolding performance, including analyses using both high-qualit
sequencing depth (>100×), which may obscure differences among preservation treatments by compensating for variations in
from depth-related compensation. Additional analyses based on downsampled Hi-C datasets would provide a more realistic as
- Hi-C sequencing was indeed done at very high coverage to ensure saturation with long-range interactions. In order to asses
figure, now named Fig. 5). These plots highlight better performance for ethanol and AllProtect treated samples, reduction of l
Note that these patterns are also reflected in scaffolding performance in most complex 10-20% of mosquito genomes represe

3. I am particularly interested in the chromosome anchoring rates observed in the Hi-C scaffolding analyses. It would be valu
methods lead to differences in chromosome anchoring efficiency, as this is a key indicator of scaffolding quality.
- We left the Hi-C scaffolded plots (now Fig. 6) exactly as they came out of a standard routine pipeline on purpose to highligh
in An. coluzzii. At this stage samples would have gone for curation, but we wanted to show which one performed well and wo
now added the % of reads that fall into a chromosomal scaffold for each PacBio and Hi-C combination in the upper right corne
span at least one chromosome arm, and showcase how much unscaffolded "shrapnel" is left.

4. The manuscript indicates that sequencing data are not yet publicly available due to their inclusion in a larger ENA project.
including expected timelines and accession numbers where possible. Furthermore, additional methodological details, particula
reproducibility and transparency.
- Since the manuscript's submission all raw data has been made available in their specific ENA study (PRJEB98990), and all p

5. In Table 1, the inclusion of ULI scaffolding appears somewhat abrupt, as ULI sequencing is not sufficiently introduced or co
Results section, readers may find it difficult to fully understand its relevance at this stage. The authors may consider introduc
table caption, to improve clarity and ensure a more coherent presentation.
- We have now added a paragraph in the Methods section, DNA extraction and long read PacBio sequencing subsection, expla
reasoning for its inclusion.

Reviewer 2:
This manuscript presents a systematic evaluation of preservation methods for generating high-quality genomic and Hi-C data
challenge in genomics, particularly for field-based sample collection where optimal preservation conditions are often difficult t
multiple preservation treatments provides useful insights for the community.
However, several aspects of the study require further clarification and improvement. In particular, concerns remain regarding
statistical analyses, and the interpretation of Hi-C results under very high sequencing coverage. Additionally, issues related to
reproducibility. Addressing these points would substantially strengthen the manuscript.
1. The study is conducted exclusively on Anopheles coluzzii under controlled laboratory conditions. While the results are prom
Given the diversity in cuticle structure, body size, and physiology across taxa, the authors should clarify the extent to which t
limitations would strengthen the manuscript.
- Answered in Reviewer 1. question 1.

2. The evaluation of Hi-C data quality is based on scaffolding performance, including analyses using both high-quality and fra

(>100×), which may mask differences in preservation efficiency. The authors should clarify how they distinguish true preserv
downsampled Hi-C data would provide a more realistic assessment of performance under typical conditions.
- Answered in Reviewer 1. question 2.

3. I am particularly interested in the chromosome anchoring rate of the genome assemblies in the Hi-C scaffolding analysis. I
preservation methods result in differences in chromosome anchoring efficiency.
- Answered in Reviewer 1. question 3.

4. The manuscript indicates that sequencing data are not yet publicly available due to their inclusion in a larger ENA project.
including expected timelines and accession numbers if available. Furthermore, greater detail in the Methods section—particula
- Answered in Reviewer 1. question 4.

5. The column headers “Self scaffolding” and “ULI scaffolding” in Table 1 are not sufficiently clear, making it difficult for reade
- We have now modified the table legend to include a more detailed description of the two column heathers, as well as a para

Additional reviewer reports:
Reviewer #1: This is a straightforward and valuable study. Demonstrating that breaking the cold chain is feasible has strong
view, the broader significance of this advance is somewhat underemphasized in the Introduction (Really just a short mention
specifically to generate genomes from very small insects, I can attest that much of arthropod biodiversity (especially within s
this. The practical importance of scalable, field-friendly preservation solutions cannot be overstated.
- Overall, the study is well designed, clearly presented, and addresses a real logistical bottleneck in biodiversity genomics. Th
that I believe would further strengthen the manuscript and increase its practical value and reproducibility.

Major / Substantive Comments
1. Framing and impact
As above, I recommend strengthening the Introduction's framing of downstream biodiversity applications, especially for smal
primary limiting factor. The method has particularly high relevance for large-scale efforts targeting hyperdiverse insect group
the paper's audience and appeal.
- We thank the reviewer for their enthusiastic comments to our manuscript. We have now added a few more sentences in the
storage and shipment methods for arthropod and dark taxa studies.

2. Practical protocol clarity and reproducibility
Because this method is likely to be adopted by field teams, including non-specialists, it would be very helpful to include a con
specimen size considerations, and timing constraints. A short practical guide or decision framework would improve reproducib
- The detailed description (with images) on how to collect and squish specimens was published separately in a protocols.io SO

Relatedly, laying out any known tolerance ranges in this fashion would strengthen the paper, for example, how sensitive outc
field conditions.
- While we have not tested this on non-Anophelines, we have since received and sequenced several Anopheles species collect
be published as genome notes in the near future. We unfortunately have not done more extensive testing on storage times a
already we are passively able to track this through possible sample failure or underperformance in the future.

3. Preservation benchmarking
I suggest including a summary comparison (table or figure) of yield/quality metrics across preservation treatments relative to
readers to interpret and apply.
- We have now added Table 3 in the Discussion section which briefly summarizes success across our storage solutions and ex
the final rating. This should make the discussion paragraph much easier to follow and for other researchers to decide on wha

4. Voucher integrity and downstream taxonomic usability
Given that many target organisms will come from taxonomically difficult groups, a short discussion of voucher integrity after
suitability for downstream taxonomic work would significantly increase the method's usefulness for specimen-based biodivers
- As this method is extremely destructive, taxonomy should be performed initially and any valuable body part (such as mouth
on assessing morphological preservation in the specified storage solutions and subsequent freezer storage. We do highlight in
and better storage of samples for morphology, proteins and metabolites, even if they were out of scope for our manuscript.

5. Methodological Clarification
The instruction to "lightly squish" specimens to compromise the cuticle is practical, but could benefit from additional detail. D

preferable (to access muscle tissue), or is abdominal disruption sufficient? This may seem like a fine detail, but it matters in p
taxonomic value. If there is an optimal or recommended compression location, it would be useful to specify it.
- As mentioned in answer 2, we have a fully detailed protocols.io SOP on how to perform the squish approach with images, w

Editor Comments:
To meet the journal's data availability requirements and ensure the reproducibility of your research findings, we kindly reques
- We have now added a Data Availability paragraph and information to each ENA raw datafile in the supplementary table. Wh
these will become available once the paper is out? We have also reformatted the manuscript to better fit the GigaScience Tec

GigaScience has also published a number of relevant papers that may help contextualize your work. We have listed several re
in the Background or Discussion—would help frame your contribution within ongoing community efforts and strengthen the p
1. Howard C, Denton A, Jackson B W, et al. On the path to reference genomes for all biodiversity: laboratory protocols and le
GigaScience, 2025, 14: giaf119.
https://urldefense.proofpoint.com/v2/url?u=https-3A__doi.org_10.1093_gigascience_giaf119&d=DwIBaQ&c=D7ByGjS34AllFgecYw0iC6Zq7qlm8uclZFI0SqQnqBo&r=fXfLn8Z4i5
zus-MsW0uJUK54GJQ6DdRQUoLo0&s=aCZk9jZoxIU3FwoX07GkNBOj-gl021vG_pEcnMYuu8k&e=
2. Lawniczak M K N, Kocot K M, Astrin J J, et al. Best-practice guidance for Earth BioGenome Project sample collection and pr
2025, 14: giaf041.
https://urldefense.proofpoint.com/v2/url?u=https-3A__doi.org_10.1093_gigascience_giaf041&d=DwIBaQ&c=D7ByGjS34AllFgecYw0iC6Zq7qlm8uclZFI0SqQnqBo&r=fXfLn8Z4i5
zus-MsW0uJUK54GJQ6DdRQUoLo0&s=G00XoY13smvkFBBI0VMkI9_-c5d-0JqyQ_0miIoGVpY&e=
- We have updated the two references now as at time of original submission they were only available as preprints.

Editorial Manager®

Cookie Preference Center

We use cookies which are necessary to make our site work. We may also use additional cookies to analyze, improve and personalize our content and your digital experience. For more information, see our Cookie Policy.

You may choose not to allow some types of cookies. However, blocking some types may impact your experience of our site and the services we are able to offer. See the different category headings below to find out more or change your settings.

Allow all

Manage Consent Preferences

Strictly Necessary Cookies

Always active

These cookies are necessary for the website to function and cannot be switched off in our systems. They are usually only set in response to actions made by you which amount to a request for services, such as setting your privacy preferences, logging in or filling in forms. You can set your browser to block or alert you about these cookies, but some parts of the site will not then work.

Functional Cookies

These cookies enable the website to provide enhanced functionality and personalisation. They may be set by us or by third party providers whose services we have added to our pages. If you do not allow these cookies then some or all of these services may not function properly.

Performance Cookies

These cookies allow us to count visits and traffic sources so we can measure and improve the performance of our site. They help us to know which pages are the most and least popular and see how visitors move around the site.

Targeting Cookies

These cookies may be set through our site by our advertising partners. They may be used by those companies to build a profile of your interests and show you relevant adverts on other sites. If you do not allow these cookies, you will experience less targeted advertising.

Cookie List

Clear

Apply Cancel

Consent Leg.Interest

Confirm my choices

Powe
