# Supplementary material for: Breaking the cold chain: solutions for room temperature preservation of mosquitoes leading to high quality reference genomes
Source: Gigascience. 2026 May 22;15:giag061. doi: 10.1093/gigascience/giag061 (PMC13221836; doi:10.1093/gigascience/giag061)
Supplement: giag061_Reviewer_1_Report_original_submission — Reviewer 1 -- 2/17/2026 [file giag061_reviewer_1_report_original_submission.pdf]

Reviewer Report

Title: Breaking the cold chain: solutions for room temperature preservation of mosquitoes leading to high quality reference genomes

Version: Original Submission **Date:** 2/17/2026

Reviewer name: Reviewer 1

Reviewer Comments to Author:

This is a straightforward and valuable study. Demonstrating that breaking the cold chain is feasible has strong potential to transform how genomes can be obtained across much of biodiversity. In my view, the broader significance of this advance is somewhat underemphasized in the Introduction (Really just a short mention around lines 72-75). Based on firsthand experience in large field campaigns designed specifically to generate genomes from very small insects, I can attest that much of arthropod biodiversity (especially within small-bodied, hyperdiverse "dark taxa") stands to benefit substantially from approaches like this. The practical importance of scalable, field-friendly preservation solutions cannot be overstated.Overall, the study is well designed, clearly presented, and addresses a real logistical bottleneck in biodiversity genomics. The conclusions are generally well supported by the data presented. I have a few suggestions that I believe would further strengthen the manuscript and increase its practical value and reproducibility.Major / Substantive Comments1. Framing and impactAs above, I recommend strengthening the Introduction's framing of downstream biodiversity applications, especially for small-bodied and taxonomically challenging groups where cold-chain logistics are often the primary limiting factor. The method has particularly high relevance for large-scale efforts targeting hyperdiverse insect groups ("dark taxa"), and this applied significance could be more explicitly highlighted to broaden the paper's audience and appeal.2. Practical protocol clarity and reproducibilityBecause this method is likely to be adopted by field teams, including non-specialists, it would be very helpful to include a concise protocol-style summary or workflow outlining the recommended handling steps, specimen size considerations, and timing constraints. A short practical guide or decision framework would improve reproducibility and uptake.Relatedly, laying out any known tolerance ranges in this fashion would strengthen the paper, for example, how sensitive outcomes are to delays in processing, temperature variation, or specimen size differences under field conditions.3. Preservation benchmarkingI suggest including a summary comparison (table or figure) of yield/quality metrics across preservation treatments relative to standard cold-chain approaches. This would make performance differences easier for readers to interpret and apply.4. Voucher integrity and downstream taxonomic usabilityGiven that many target organisms will come from taxonomically difficult groups, a short discussion of voucher integrity after treatment would be valuable. Guidance on expected morphological preservation and suitability for downstream taxonomic work would significantly increase the method's usefulness for specimen-based biodiversity genomics workflows.Methodological ClarificationThe instruction to "lightly squish" specimens to compromise the cuticle is practical, but could benefit from additional detail. Does the location of compression affect outcomes? For example, is thoracic compression preferable (to access muscle tissue), or is abdominal disruption sufficient? This

may seem like a fine detail, but it matters in practice, particularly because damage to thoracic characters or terminalia can reduce taxonomic value. If there is an optimal or recommended compression location, it would be useful to specify it.

Level of Interest

Please indicate how interesting you found the manuscript: Choose an item.

Quality of Written English

Please indicate the quality of language in the manuscript: Choose an item.

Declaration of Competing Interests

Please complete a declaration of competing interests, considering the following questions:

- Have you in the past five years received reimbursements, fees, funding, or salary from an organisation that may in any way gain or lose financially from the publication of this manuscript, either now or in the future?
- Do you hold any stocks or shares in an organisation that may in any way gain or lose financially from the publication of this manuscript, either now or in the future?
- Do you hold or are you currently applying for any patents relating to the content of the manuscript?
- Have you received reimbursements, fees, funding, or salary from an organization that holds or has applied for patents relating to the content of the manuscript?
- Do you have any other financial competing interests?
- Do you have any non-financial competing interests in relation to this paper?

If you can answer no to all of the above, write 'I declare that I have no competing interests' below. If your reply is yes to any, please give details below.

I declare that I have no competing interests

I agree to the open peer review policy of the journal. I understand that my name will be included on my report to the authors and, if the manuscript is accepted for publication, my named report including any attachments I upload will be posted on the website along with the authors' responses. I agree for my report to be made available under an Open Access Creative Commons CC-BY license (http://creativecommons.org/licenses/by/4.0/). I understand that any comments which I do not wish to be included in my named report can be included as confidential comments to the editors, which will not be published.

I agree to the open peer review policy of the journal

To further support our reviewers, we have joined with Publons, where you can gain additional credit to further highlight your hard work (see: https://publons.com/journal/530/gigascience). On publication of this paper, your review will be automatically added to Publons, you can then choose whether or not to claim your Publons credit. I understand this statement.

Yes Choose an item.
